# Heteroallene Insertions into Tin(II) Alkoxide Bonds

**DOI:** 10.1021/acs.inorgchem.3c04551

**Published:** 2024-06-04

**Authors:** Aidan T. Ryan, Andrew Brookes, Andrew J. Straiton, Thomas Wildsmith, John P. Lowe, Kieran C. Molloy, Michael S. Hill, Andrew L. Johnson

**Affiliations:** † Department of Chemistry, University of Bath, Claverton Down, Bath BA2 7AY, United Kingdom; ‡ Center for Sustainable Chemical Technologies, University of Bath, Bath BA2 7AY, United Kingdom; § Material and Chemical Characterisation Facility (MC^2^), University of Bath, Bath BA2 7AY, United Kingdom

## Abstract

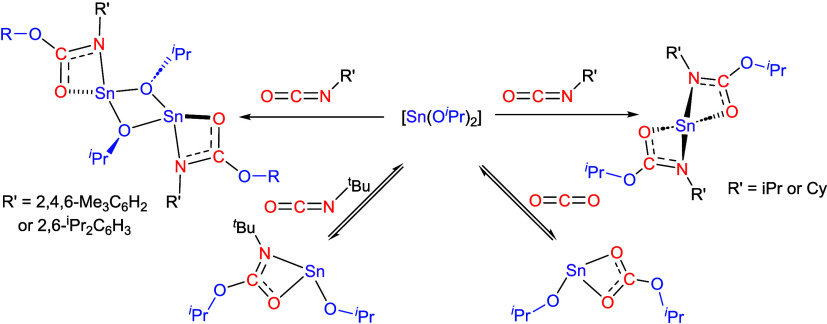

A series of iso-carbamate complexes have been synthesized by the reaction of [Sn^II^(O^*i*^Pr)_2_] or [Sn^II^(O^*t*^Bu)_2_] with either aryl or alkyl isocyanates, ONC-R (R = 2,4,6-trimethylphenyl (Mes), 2,6-diisopropylphenyl (Dipp), isopropyl (^i^Pr), cyclohexyl (Cy) and *tert*-butyl (^t^Bu)). In the case of aryl isocyanates, mono-insertion occurs to form structurally characterized complexes [Sn{κ^2^-*N,O*-R-NC(O^*i*^Pr)O}(μ-O^*i*^Pr)]_2_ (**1**: R = Mes, **2**: R = Dipp) and [Sn{κ^2^-*N,O*-R-NC(O^*t*^Bu)O}(μ-O^*t*^Bu)]_2_ (**3**: R = Mes, **4**: R = Dipp). The complicated solution-state chemistry of these species has been explored using ^1^H DOSY experiments. In contrast, reactions of tin(II) alkoxides with alkyl isocyanates result in the formation of bis-insertion products [Sn{κ^2^-*N,O*-R-NC(O^i^Pr)O}_2_] (**5**: R = ^*i*^Pr, and **6**: R = Cy) and [Sn{κ^2^-*N,O*-R-NC(O^t^Bu)O}_2_] (**7**: R = ^*i*^Pr, **8**: R = Cy), of which complexes **6**–**8** have also been structurally characterized. ^1^H NMR studies show that the reaction of ^*t*^Bu-NCO with either [Sn(O^*i*^Pr)_2_] or [Sn(O^*t*^Bu)_2_] results in a reversible mono-insertion. Variable-temperature 2D ^1^H–^1^H exchange spectroscopy (VT-2D-EXSY) was used to determine the rate of exchange between free ^*t*^Bu-NCO and the coordinated ^*t*^Bu-iso-carbamate ligand for the {O^*i*^Pr} alkoxide complex, as well as the activation energy (*E*_a_ = 92.2 ± 0.8 kJ mol^–1^), enthalpy (Δ*H*^‡^ = 89.4 ± 0.8 kJ mol^–1^), and entropy (Δ*S*^‡^ = 12.6 ± 2.9 J mol^–1^ K^–1^) for the process [Sn(O^*i*^Pr)_2_] + ^*t*^Bu-NCO ↔ [Sn{κ^2^-N,O-^*t*^Bu-NC(O^*i*^Pr)O}(O^*i*^Pr)]. Attempts to form Sn(II) alkyl carbonates by the insertion of CO_2_ into either [Sn(O^*i*^Pr)_2_] or [Sn(O^*t*^Bu)_2_] proved unsuccessful. However, ^119^Sn{^1^H} NMR spectroscopy of the reaction of excess CO_2_ with [Sn(O^*i*^Pr)_2_] reveals the presence of a new Sn(II) species, i.e., [(^*i*^PrO)Sn(O_2_CO^*i*^Pr)], VT-2D-EXSY (^1^H) of which confirms the reversible alkyl carbonate formation (*E*_a_ = 70.3 ± 13.0 kJ mol^–1^; Δ*H*^‡^ = 68.0 ± 1.3 kJ mol^–1^ and Δ*S*^‡^ = −8.07 ± 2.8 J mol^–1^ K^–1^).

## Abstract 2

A series of mono- or bis-iso-carbamate Sn(II) complexes have been synthesized by the reaction of [Sn^II^(O^*i*^Pr)_2_] or [Sn^II^(O^*t*^Bu)_2_] with heteroallenes, i.e., aryl and alkyl isocyanates, ONC-R (R = 2,4,6-trimethylphenyl (Mes), 2,6-diisopropylphenyl (Dipp), isopropyl (^i^Pr), cyclohexyl (Cy), *tert*-butyl (*^t^*Bu)) and CO_2_. The degree to which the parent alkoxides undergo either mono- or bis-insertion into the heteroallene group has been explored both experimentally and computationally. The mechanism of aryl and alkyl isocyanate insertion and CO_2_ insertion into {Sn–OR} bonds are discussed.

## Introduction

The insertions of unsaturated polar heteroallene molecules, E^δ−^ = C^δ+^ = X^δ−^ (E, X = O, S or N–R), into M–CR_3_, M–NR_2_, and M–OR bonds represent a fundamental step in metal-promoted transformations. Relevant to catalysis for the formation of new bonds in which heteroallenes (e.g., CO_2_, OCS, CS_2_, OCN-R, SCN-R) may be converted into added-value chemicals,^1^ such reactivity also provides a straightforward route to the preparation of novel complexes with potential capability to serve as precursors in catalysis^2^ and materials science.^3^ While the vast majority of interest in this type of reaction has primarily focused on the insertion of carbon dioxide, isocyanates, and carbodiimides into transition metal M–C and M–N bonds, there has recently been a surge in the use of s and p block metals and metalloids to achieve similar ends.

These insertion reactions are strongly dependent on both the electrophilicity of the central C^δ+^ of the heteroallene and the nucleophilicity of the M–C^δ−^, M–N^δ−^, and M–O^δ−^ bonds. Isocyanates are known to be highly reactive electrophiles, and CO_2_ is generally considered to be a rather inert molecule, with the insertion into M–N and M–O bonds controlled by thermodynamic factors.^1a,4^

**Figure 1:**
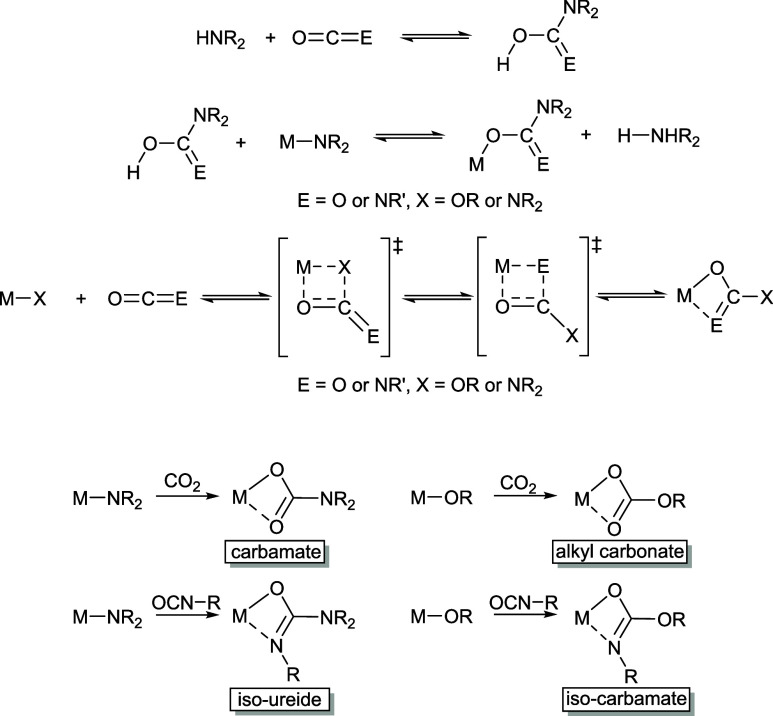
Possible insertion mechanisms of heteroallenes O=C=E (E = O or NR’) into M–NR_2_ or M–OR bonds. The formation of carbamate, alkyl carbonate, iso-ureide, and iso-carbamate systems from the reaction of metal amides and metal alkoxides with CO_2_ and isocyanates.

Two possible mechanisms for heteroallene insertion into M–N bonds have been postulated: First, insertion may occur via a four-membered transition state (e.g., Figure 1) in which the amide or alkoxide ligand acts as a nucleophile toward the electrophilic carbon atom concurrent with coordination of one of the oxygen atoms to the metal center and a weakening of the M–N/O and the C=O bonds (Figure 1). For this mechanism, it is generally accepted that an open coordination site at the metal center is unnecessary for such nucleophilic insertion reactions, with the reaction proceeding via initial interaction of the Lewis acidic carbon (C^δ+^) of the heteroallene with the lone pairs on the alkoxide oxygen or amide nitrogen, followed by a concerted transformation to a four-centered transition state (Figure 1).^5^ The kinetic facilitator for this reaction is generally thought to be the strong nucleophilicity of the alkoxide or amide ligand.

A second possible mechanism suggests that in the case of CO_2_ insertion into M–N bonds of metal amide complexes proceeds via an amine-catalyzed process, according to a generalized mechanism for an A = B dipole into an M–N bond shown below (Figure 1), rather than a direct insertion mechanism.^6^

While both are plausible mechanisms, there is no prima facie evidence, indicating that metal alkoxides undergo similar reactions with either CO_2_ or isocyanates.

While CO_2_ and isocyanates have commonly been observed to be inserted into the M–N bond of metal amide complexes to form carbamates and iso-ureides, respectively (Figure 1), the comparable reaction between metal alkoxides with CO_2_ and isocyanates to form alkyl carbonates and iso-carbamates, respectively, has been significantly less studied, with metal–alkoxide complexes showing either low or reversible reactivity with CO_2_.

The reactivity of metal alkoxides with heteroallenes X=C=E (X, E = O, S, NR) allows for the formation of many potential derivatives, i.e., metal carbamates, metal iso-ureides, metal alkyl carbonates, and metal iso-carbamates, that can be evaluated as precursors for the formation of metal-based thin films via chemical vapor deposition (CVD). While the use of metal carbamates, [M{O_2_CNR_2_}*_n_*], and metal dithiocarbamates, [M{S_2_CNR_2_}*_n_*], in thin film deposition is extensive,^7^ the application of iso-carbamate compounds [M{RNC(O)OR}*_n_*], in CVD applications is limited to the work by Devi and co-workers who successfully deposited HfO_2_ at 250 °C with [Hf{^i^PrNC(O)O^i^Pr}_4_] at half the temperature of pre-existing HfO_2_ precursors.^8^ Similarly, the utility of metal iso-ureide, [M{RNC(O)NR_2_}*_n_*], and metal iso-thioureide, [M{RNC(S)NR_2_}*_n_*], compounds in thin film formation are currently limited to the Sn(II) bis(ureide) derivatives, [Sn{RNC(O)NMe_2_}_2_] (R = *^t^*Bu and Ad),^3k^ and [Sn(μ-NMe_2_){RNC(S)NMe_2_}]_2_^3a^ as precursors to the formation of phase pure SnO and SnS by aerosol-assisted chemical vapor deposition (AACVD), respectively.

Xanthate systems [M{S_2_COR}*_n_*] (R = alkyl) and their Lewis-base adducts formed by the insertion of CS_2_ into metal–alkoxide (M–OR) bonds have been successfully used as single-molecule or single-source precursors (SSPs) for metal sulfide material synthesis.^3e−3g,3i^ Contrastingly, the utilization of metal alkyl carbonate compounds, [M{O_2_COR}*_n_*] (R = alkyl), and their Lewis-base adducts as CVD precursors has not been described to date.

With respect to insertion chemistry, Sn(II)–OR systems are considered to have a low Lewis basicity and are hence weak nucleophiles.^9^ As such, outside the arena of lactide polymerization studies,^10^ the chemistry of tin(II) alkoxides has been little studied. In contrast, tin(IV) alkoxide systems are sufficiently nucleophilic to react with electrophiles and heteroallenes such as CO_2_ and isocyanates.^2a,11^

Fulton et al. previously reported the insertion of CO_2_ into the Sn–OR bonds of bulky β-diketiminate-supported Sn(II) alkoxide complexes, [{BDI}Sn–OR] (R = ^*i*^Pr or ^*t*^Bu; BDI = [{N(2,6-^i^Pr_2_C_6_H_3_)C(Me)}_2_CH]), which display variable reactivity depending upon the steric bulk of the alkoxide ligand.^9b^ Attempts to react phenyl isocyanate or CS_2_ with [{BDI}Sn–OR] (R = ^*i*^Pr, ^*s*^Bu or ^*t*^Bu) were inconclusive.^9b^ In sharp contrast to the Sn(II) complexes, the Ge(II) analogue^12^ shows no discernible reactivity toward CO_2_, whereas the Pb(II) analogues^12^ display reversible insertion into the Pb–OR bonds, the degree of which is highly dependent on the nature of the alkoxide group. In contrast to CO_2_ insertion into divalent group 14 metal–alkoxide bonds, insertion into either alkyl or aryl isocyanates is limited to a single example, specifically the insertion of phenyl isocyanate into the Pb–O bond of [{BDI}Pb–O^*i*^Pr] to form the iso-carbamate system [{BDI}Pb-OC(NPh)O^*i*^Pr].^12b^

As part of continuing interest in tetrylene chemistry [R_2_E] (E = Ge Sn or Pb) and the development of precursors for the deposition of thin films of both SnO^3k,13^ and SnS,^3a−3c^ we describe here the synthesis and characterization of tin(II) iso-carbamate and alkyl carbonate compounds through the insertion reactions of organic isocyanates and CO_2_, respectively, into the Sn–OR bond of tin(II) alkoxides.

## Results and Discussion

### Insertion of Aryl Isocyanates into [Sn(O^*i*^Pr)_2_] and [Sn(O^*t*^Bu)_2_]

**Scheme 1:**
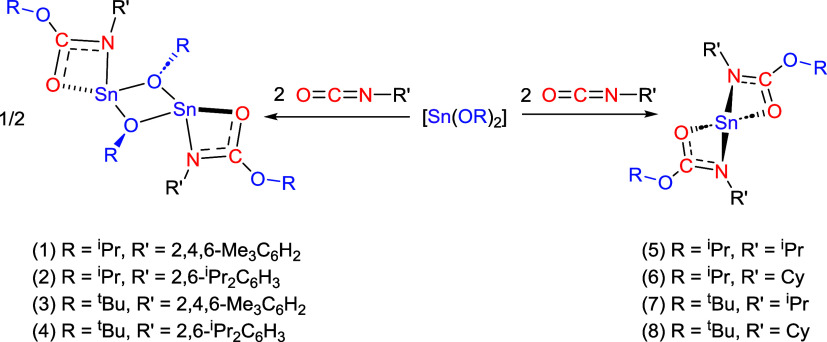
Synthesis of Sn(II) Iso-carbamate Complexes **1**–**8** via the Mono-insertion of Aryl Isocyanates (**1**–**4**) or the Bis-insertion of Alkyl Isocyanates (**5**–**8**) into Sn–OR Bonds

Addition of 2 equiv of the relevant aryl isocyanate, 2,6-diisopropylphenyl isocyanate, and 2,4,6-trimethylphenyl isocyanate to toluene solutions of tin(II) alkoxides [Sn(O^*i*^Pr)_2_] and [Sn(O^*t*^Bu)_2_], respectively, yielded mono-insertion products **1**–**4**, which were purified through crystallization from toluene (Scheme 1). The identity of the products as the heteroleptic mono-insertion species, despite the reaction stoichiometry, was established through both microanalysis and NMR spectroscopy and finally confirmed by X-ray crystallography. In all cases, multinuclear NMR studies were performed on isolated and dried crystalline products rather than reaction mixtures.

**Figure 2:**
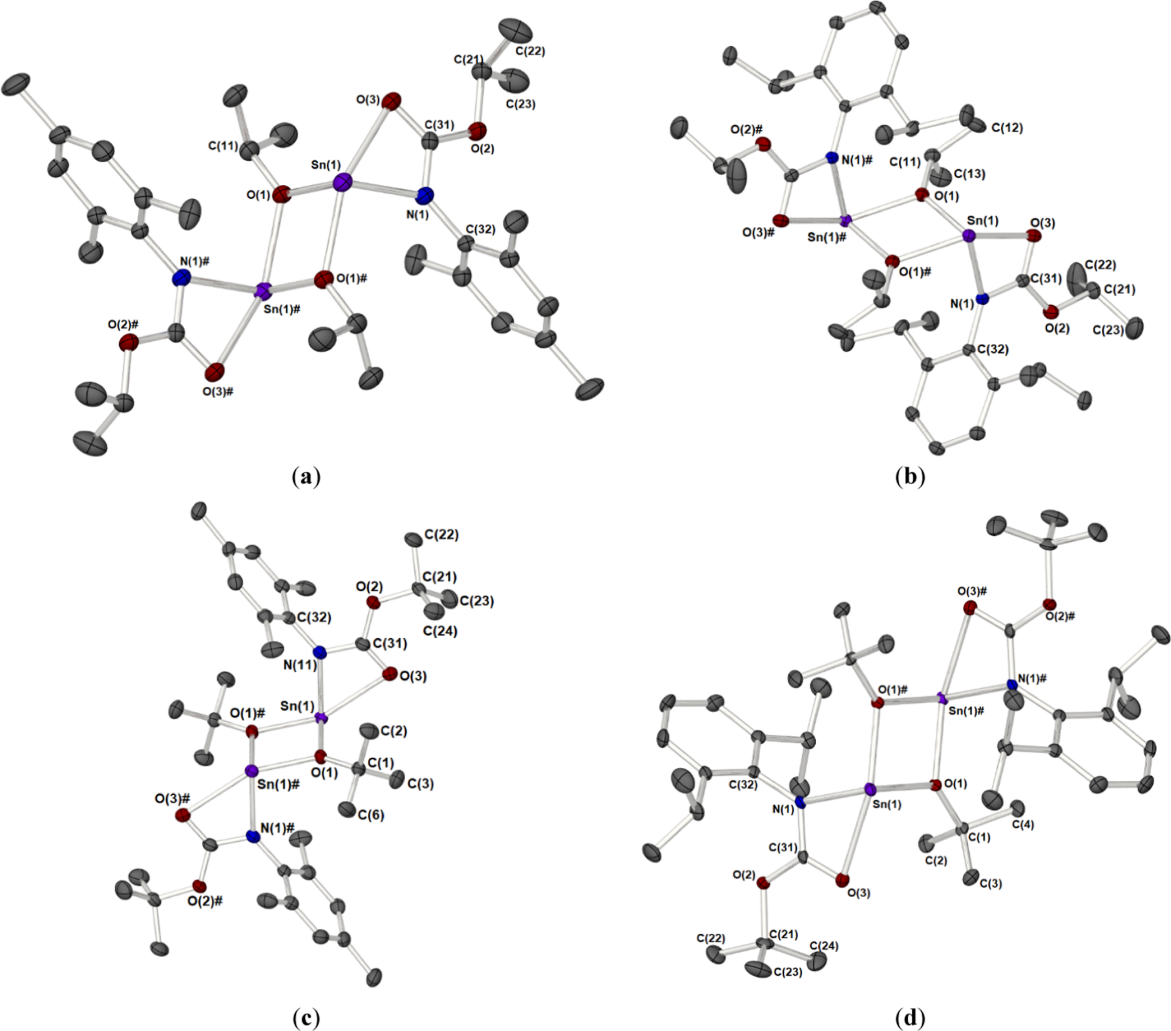
Solid-state molecular structures of complexes **1** (a), **2** (b), **3** (c), and **4** (d). In all cases, hydrogen atoms have been omitted for clarity, and thermal ellipsoids are shown at 50% probability. For complex **4**, solvent of crystallization (toluene) has been omitted for clarity. For complexes **2** and **4** (b, d), only the molecules based around the Sn(1) atoms are shown. The second molecule in each asymmetric unit cell is identical, within experimental error, and omitted for clarity. Symmetry transformations are used to generate equivalent atoms (#); **1**: 1 – *X*, 1 – *Y*, 1 – *Z*; **2**: −*X*, 2 – *Y*, −*Z*; **3**: −*X*, −*Y*, −*Z*; **4**; 2 – *X*, 1 – *Y*, 1 – *Z*.

Confirmation of the absolute molecular structures of the insertion products (**1**–**4**) was established by single-crystal X-ray diffraction analysis. The solid-state molecular structures of **1**–**4** are shown in Figure 2; a selection of bond lengths and angles can be found in Table 1.

**Table 1:** Selected Bond Lengths and Bond Angles for Compounds **1**–**4**

selected bond lengths (Å)				
	**1**	**2**	**3**	**4**
Sn(1)–O(1)	2.1293(14)	2.1202(10)	2.1372(10)	2.1406(14)
Sn(1)–O(1)#1	2.2085(13)	2.1925(10)	2.2028(10)	2.2023(16)
Sn(1)–N(1)	2.1839(15)	2.2035(12)	2.2035(13)	2.1925(19)
Sn(1)–O(3)	2.5328(14)	2.5074(11)	2.613(1)	2.582(2)
O(1)–C(11)	1.444(2)	1.4479(17)	1.468(4)	1.488(2)
N(1)–C(31)	1.237(2)	1.3301(17)	1.338(4)	1.343(3)
N(1)–C(32)	1.428(2)	1.4287(17)	1.4336(19)	1.419(3)
O(2)–C(31)	1.343(2)	1.3439(17)	1.3468(17)	1.339(3)
O(2)–C(21)	1.465(2)	1.4690(17)	1.4711(17)	1.488(3)
O(3)–C(31)	1.253(2)	1.2570(17)	1.246(2)	1.244(3)

selected bond angles (deg)				
	**1**	**2**	**3**	**4**
O(1)–Sn(1)–N(1)	95.49(6)	97.24(4)	98.59(5)	94.14(6)
O(1)–Sn(1)–O(1)#1	70.99(5)	71.34(4)	72.20(4)	73.26(6)
N(1)–Sn(1)–O(1)#1	87.89(5)	89.29(4)	93.49(4)	90.97(6)
O(1)–Sn(1)–O(3)	85.82(5)	88.59(4)	92.37(4)	93.61(6)
N(1)–Sn(1)–O(3)	55.90(5)	56.09(4)	54.62(4)	55.00(6)
O(1)#1-Sn(1)–O(3)	135.15(5)	137.90(4)	142.81(4)	144.19(6)
C(11)–O(1)–Sn(1)	129.99(12)	131.74(9)	127.07(9)	127.8(1)
C(31)–N(1)–C(32)	123.15(15)	123.92(12)	119.97(2)	124.3(1)
C(31)–N(1)–Sn(1)	99.16(11)	97.49(9)	100.2(1)	100.1(1)
C(31)–O(2)–C(21)	117.36(14)	116.71(11)	122.3(1)	121.0(2)
C(31)–O(3)–Sn(1)	85.18(11)	85.64(8)	83.86(9)	84.9(1)
O(3)–C(31)–N(1)	119.75(17)	119.14(13)	120.3(1)	119.2(2)
Sn(1)–O(1)–Sn(1)#1	109.01(5)	108.66(4)	107.91(1)	106.74(7)

The structures of compounds **1**–**4** were all found to adopt a dinuclear arrangement where each Sn(II) metal center adopts a tetra-coordination environment, comprised of a κ^2^-*O,N* chelating iso-carbamate ligand and two μ-OR (R = ^*i*^Pr or ^*t*^Bu) groups. This is in direct contrast to the polymeric solid-state structure of [Sn(O^*i*^Pr)_2_]_*x*_^14^ and is more closely related to the discrete dimer formation of [Sn(O^*t*^Bu)_2_]_2_ in the solid state.^15^ Contrastingly, in the solution state, [Sn(O^*i*^Pr)_2_] and [Sn(O^*t*^Bu)_2_] are discrete dimers.^14^

The distribution of the ligands around each Sn(II) centers can be described as possessing a distorted disphenoidal geometry.

In all cases, the compounds possess a transoidal configuration, with respect to the orientation of iso-carbamate ligands across the {Sn_2_O_2_} core. The N-substituents of the iso-carbamate ligands are thus also located on opposite sides to minimize steric interactions.

A comparison of the metrical data for **1**–**4** shows a notable asymmetry to the two Sn–μ–OR bonds involved with the bridging alkoxide groups (Table 1) in which one of the Sn–O bonds within the {Sn_2_O_2_} core is longer than the other. Similar distinctly different Sn(II)–O distances have been observed in related hypervalent stannylenes, reflecting the different O-donor ability, the geometric constraints, and electronic saturation of tin.^16^

The iso-carbamate ligands are bound to the tin(II) centers more strongly through the nitrogen with the Sn(1)–N(1) bond [2.1839(15)–2.2035(13) Å] notably shorter than the Sn(1)–O(3) bond lengths [2.5074(11)–2.613(1) Å]. The Sn(1)–O(3) bond length is found to be longest for compounds **3** and **4**, which can be attributed to the increased steric bulk on the bridging alkoxide groups, which may push the carbonyl oxygen of the iso-carbamate away from the tin(II) center.

The orientation of the OR group at the back of the iso-carbamate ligand is of interest, as this can be indicative of the electron delocalization from the OR moiety into the {N–C–O} backbone, with delocalization favored when the OR group is coplanar with the N–C–O backbone. For all four compounds, the OR group is orientated approximately coplanar to the N–C–O backbone with the maximum deviation of 8° {O(3)–C(31)–O(2)-C(21)} measured for compound **3**. The delocalization is reflected for all four compounds with the C(31)–O(2) bond length [1.339(2)-1.3468(17) Å] being ca. 0.1 Å shorter than the C(21)–O(2) bond [1.465(2)–1.488(3) Å].

The ^1^H NMR spectrum of **1** displays two distinct {CH} resonances at δ = 4.67 and 5.42 ppm, corresponding to the two different {O^*i*^Pr} groups, and resonances related to the associated methyl groups of the {O^*i*^Pr} groups at δ = 1.35 and 1.41 ppm. In the corresponding region of the ^13^C{^1^H} NMR spectrum, four resonances can be assigned to the methyl groups of the different {O^*i*^Pr} units, alongside two methyl resonances arising from the methyl substituents on the aryl ring. Two ^13^C{^1^H} NMR resonances (δ = 69.0, 69.1 ppm) were assigned to the two methine environments of the iso-carbamate and alkoxide {O^*i*^Pr} groups. A resonance at δ = 161.9 ppm in the ^13^C{^1^H} NMR spectrum is diagnostic of the {NC(O)O} backbone carbon, while integration of the signals due to the methyl groups of the aliphatic and aromatic components (^13^C{^1^H} NMR) is also consistent with a mono-insertion product. A single resonance in the ^119^Sn{^1^H} NMR spectrum at −313 ppm is close to the resonances observed for the 4-coordinate tin(II) bis-ureate compounds we have reported elsewhere^13e^ and consistent with the presence of a dimeric species in solution.

The ^1^H NMR spectrum for **2** shows the heteroleptic nature of the compound in a clearer fashion with four doublet resonances observed, two for the {^*i*^Pr} substituents on the aryl group (δ 1.39 and 1.42 ppm; both ^3^*J* = 10 Hz) and two for the alkoxide and iso-carbamate isopropyl groups (δ 1.07 ppm (^3^*J* = 5 Hz) and 1.14 ppm (^3^*J* = 10 Hz), respectively). Although only three methine resonances were observed, the multiplet at δ 3.69–3.77 ppm integrated for 2H atoms, suggesting an overlap of the signals arising from the {^*i*^Pr} methine groups bound to the aryl substituent. Further septet resonances (δ 4.34, 5.11 ppm) arise from the {CH} protons of the two distinct O^*i*^Pr environments, while a multiplet at 7.05–7.21 ppm could be assigned to the aromatic protons. In the ^13^C{^1^H} NMR spectrum of **2**, there are four resonances corresponding to {^*i*^Pr} methyl groups at δ 22.6–29.4 ppm for the alkoxide, iso-carbamate, and aryl substituents. Two resonances at δ 69.1 and 69.7 ppm assigned to the methine carbons suggested some overlap for both the aryl substituents and the alkoxide and iso-carbamate ^*i*^Pr groups. A further four resonances for the aromatic carbons were observed at δ 123.7–145.5 ppm, while the {NC(O)O} backbone carbon provided a low-field resonance at δ 162.4 ppm. Compound **2** exhibits a single resonance in its ^119^Sn{^1^H} NMR spectrum at δ −345 ppm in solution, consistent with **1**.

As part of our study, ^1^H DOSY NMR experiments were undertaken using the external calibration curve (ECC) approach, with temperature-independent normalized diffusion coefficients, for MW determination described elsewhere.^17^Table 2 shows the experimentally determined diffusion coefficients at both 298 and 350 K in C_6_D_6_.

**Table 2:** Experimentally Determined (^1^H DOSY NMR) Diffusion Coefficients (*D*_Exp_), Calculated Hydrodynamic Radii (*R*_Hyd-cal_), and Calculated Molecular Weight (MW_cal_) of **1**–**4** at Temperatures 298 and 350 K alongside Molecular Weight (MW), Molecular Volume (*V*_Xray_), and Molecular Radius (*R*_Xray_) as Determined from Single-Crystal X-ray Diffraction

	298 K			350 K			solid state		
	*D*_Exp_ (m^2^ s^–1^)	*R*_Hyd-cal_ (Å)	MW_cal_ (g/mol)	*D*_Exp_ (m^2^ s^–1^)	*R*_Hyd-cal_ (Å)	MW_cal_ (g/mol)	MW (g/mol)	*V*_Xray_ (Å^3^)	*R*_Xray_^a^ (Å)
**1**	5.62 × 10^–10^	6.32	991	1.28 × 10^–9^	6.36	1009	798.2	566.40	5.13
**2**	6.05 × 10^–10^	5.87	840	1.27 × 10^–9^	5.48	1027	882.3	655.01	5.39
**3**	6.33 × 10^–10^	5.61	760	1.48 × 10^–9^	5.51	730	854.2	631.71	5.32
	1.32 × 10^–9^	2.69	166	2.81 × 10^–9^	2.08	192			
**4**	6.68 × 10^–10^	5.32	675	2.4 × 10^–9^	3.40	263	938.3	713.66	5.43
	1.06 × 10^–9^	3.35	256						

a Calculation assumes a spherical molecule.

^1^H DOSY NMR studies of complexes **1** and **2** at both 298 and 350 K show single diffusion coefficients, corresponding to hydrodynamic radii of ca. 6.3–6.4 and 5.5–5.9 Å, respectively, across this temperature range. These values are consistent with molecular radii of complexes **1** and **2** (5.13 and 5.39 Å) based on the single-crystal data, i.e., molecular volumes of 566 and 655 Å^3^, respectively.

While approximate relationships between the molecular weights of small molecules (<1000 g mol^–1^) containing light atoms and their diffusion coefficients are known, they are somewhat unreliable for species containing heavier atoms (larger than sulfur). Using the previously reported Stokes–Einstein–Gierer–Wirtz Estimation (SEGWE) calculator reported by Evans et al.,^18^ we have estimated the calculated molecular weights of the solution-state species (Table 2). Even considering errors associated with calculated molecular weights from diffusion coefficients, our observations point to the presence of dimeric species in solution, at both 298 and 350 K, to be commensurate with those observed in the solid state.

The reaction of [Sn(O^*t*^Bu)_2_] with mesityl isocyanate and 2,6-diisopropylphenyl isocyanate, separately, followed by recrystallization, results in the formation of two sets of colorless crystals. Analysis by ^1^H NMR spectroscopy (C_6_D_6_) reveals spectra for **3** and **4**, respectively, to be complicated and present multiple {^*t*^BuO}, {^*i*^Pr}, and {Me} environments, consistent with the presence of multiple species in the solution state. This is supported by the ^119^Sn{^1^H} (C_6_D_6_) NMR spectra for **3** and **4**: At 298 K, the ^119^Sn{^1^H} spectrum of **3** (in C_6_D_6_) shows three resonances at −99, −273, and −295 ppm. In contrast, the ^119^Sn{^1^H} spectrum of **4** (in C_6_D_6_) at 298 K shows a single resonance at −103 ppm.

Warming NMR samples of **3** and **4** to 373 K in *d*_8_-Tol results in a change in the appearance of both the ^1^H and ^13^C{^1^H} NMR spectra, which show evidence of coalescence resulting in a simplified spectrum, consistent with a mono-insertion product. For **3**, at 373 K, two broad singlet resonances in the ^1^H and ^13^C{^1^H} spectra (^1^H δ: 2.02 ppm, 2.03 ppm, ^13^C δ: 34.2 ppm, 34.9 ppm) corresponding to the methyl groups of the {^*t*^Bu} moieties of the alkoxide and iso-carbamate ligands, respectively, are observed. This, alongside two distinct quaternary carbon atoms in the ^13^C spectrum (^13^C δ 74.3, 75.1 ppm) and a resonance at 165.8 ppm, confirms the formation of the iso-carbamate system. Similar observations were made for complex **4**, with resonances at 373 K in *d*_8_-Tol, and the ^1^H NMR spectrum (a single broad resonance at δ = 1.41 ppm corresponding to the methyl groups of the {^*t*^Bu} moieties of the alkoxide and iso-carbamate ligands) and the ^13^C{^1^H} NMR spectrum (two distinct quaternary carbon atoms, δ 74.2 and 76.0 ppm, and a resonance at 164.7 ppm) again confirm the formation of the iso-carbamate system. ^1^H and ^119^Sn{1H} NMR spectra of **3** and **4** were also recorded in C_6_D_6_ at 350 K and showed identical chemical shifts to those performed in *d*_8_-Tol at 373 K.

In contrast to the ^119^Sn{^1^H} spectra of **3** and **4**, at 298 K, those recorded at either 350 K (in C_6_D_6_) or 373 K in *d*_8_-Tol show resonances at δ = −100 and −104 ppm (**3**) and δ = −100 ppm (**4**). We believe the resonance observed at δ = −100 ppm is consistent with the presence of [Sn(O^*t*^Bu)_2_] and the small change in chemical shift compared to [Sn(O^*t*^Bu)_2_] at 298 K (δ = −94 ppm)^19^ to be due to solvent effects. While the precise identities of the species in the ^119^Sn{^1^H} spectra of **3** and **4**, at both 298 and 350 K, are unknown, we speculate that the presence of [Sn(O^*t*^Bu)_2_] is the result of partial deinsertion of aryl isocyanate from the dimeric insertion products observed in the solid state.

**Scheme 2:**
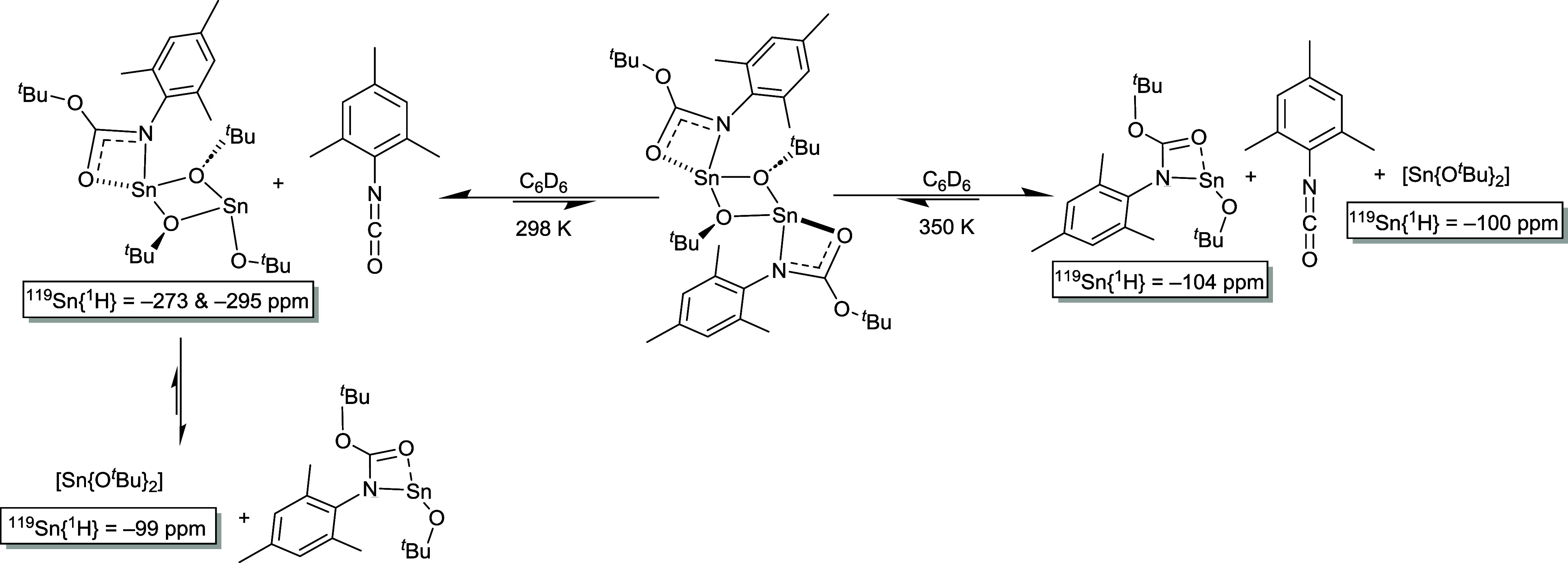
Scheme Highlighting the Proposed Identities (and Associated ^119^Sn NMR Resonances) of the Postulated Species Observed in the Solution State at 298 and 350 K

To assess this, ^1^H DOSY NMR experiments at both 298 and 350 K were performed. Table 2 shows the experimentally determined diffusion coefficients for **3** and **4** at both 298 and 350 K in C_6_D_6_. ^1^H DOSY NMR studies of complex **3** at these temperatures show, in each case, two distinct diffusion coefficients, which correspond to hydrodynamic radii of ca. 5.61 and 2.69 Å, at 298 K, and ca. 5.51 and 2.08 Å, at 350 K, respectively: In both cases, the species with smaller diffusion coefficients and larger hydrodynamic radii (*R*_Hyd_) are consistent with the experimentally determined crystallographic radius of 5.32 Å for **3** (MW = 854 g/mol), with SEGWE-calculated molecular masses (MW_Cal_) of ca. 760 and 730 g/mol, both lower than that expected for complex **3** (MW = 854.3 g/mol). The second species observed in the solution with larger diffusion coefficients has calculated *R*_Hyd_ values of 2.69 Å (298 K) and 2.08 Å (350 K). As can be seen in Table 2, the SEGWE-calculated molecular masses (MW_Cal_) of these two species are ca. 166 and 192 g/mol, which we suggest is attributable to free mesityl isocyanate (MW = 161.08 g/mol). Allowing for a significant experimental error, we speculate that in the case of complex **3** in solution, at both 298 and 350 K, deinsertion of mesityl isocyanate occurs: At 298 K, deinsertion results in formation of [{κ^2^-O,O′,-Mes-NC(O^*i*^Pr)O}Sn(μ^2^-O*^t^*Bu)_2_Sn(O*^t^*Bu)] (^119^Sn{^1^H}: δ = −273 and −295 ppm), which is in exchange with [{κ^2^-O,O′,-Mes-NC(O^*i*^Pr)O}Sn(O^t^Bu)] (consistent with the SEGWE-calculated molecular mass, i.e., 760 g/mol vs MW = 693 g/mol) and [Sn(O^t^Bu)_2_] (^119^Sn{^1^H}: δ = −99 ppm), as shown in Scheme 2. At 350 K, deinsertion of mesityl isocyanate results in formation of [{κ^2^-O,O′,-Mes-NC(O^*i*^Pr)O}Sn(O*^t^*Bu)] (^119^Sn{^1^H}: δ = −104 ppm) free mesityl isocyanate and [Sn(O^t^Bu)_2_] (^119^Sn{^1^H}: δ = −104 ppm), as shown in Scheme 2.

^1^H DOSY of **4** at 298 K yields results similar to those of **3**, from which we can draw comparable conclusions. However, as already noted, the ^119^Sn{^1^H} spectrum of **4** shows only one resonance at −103 ppm. At elevated temperatures (350 K), 1H DOSY of **4** shows only one diffusion coefficient, which corresponds to the hydrodynamic radius of 3.4 Å and a SEWEG-calculated molecular mass of 263 g/mol.

Despite the solution-state complexities, elemental analyses for **1**–**4** are all consistent with formation of the mono-insertion products identified in the solid state.

### Insertion of Alkyl Isocyanates into [Sn(O*^i^*Pr)_2_] and [Sn(O*^t^*Bu)_2_]

In contrast to the reaction of tin(II) alkoxides with aryl isocyanates, the reaction with various alkyl isocyanates, irrespective of the ratio (i.e., 1:1 or 1:2), generated bis-insertion products, i.e., homoleptic tin(II) iso-carbamates (Scheme 1).

Products were identified on the basis of microanalysis and NMR spectroscopy and subsequently confirmed by crystallography in the case of **6**–**8**. The ^1^H/^13^C NMR spectra of **5**–**8** are unexceptional and contain resonances due solely to the iso-carbamate ligand, including a ^13^C signal at ca. 160 ppm due to the newly formed quaternary {C=O} moiety and the absence of any signals associated with alkoxide groups. Furthermore, the ^119^Sn NMR spectra contain one signal in the range of −335 to −360 ppm.

**Figure 3:**
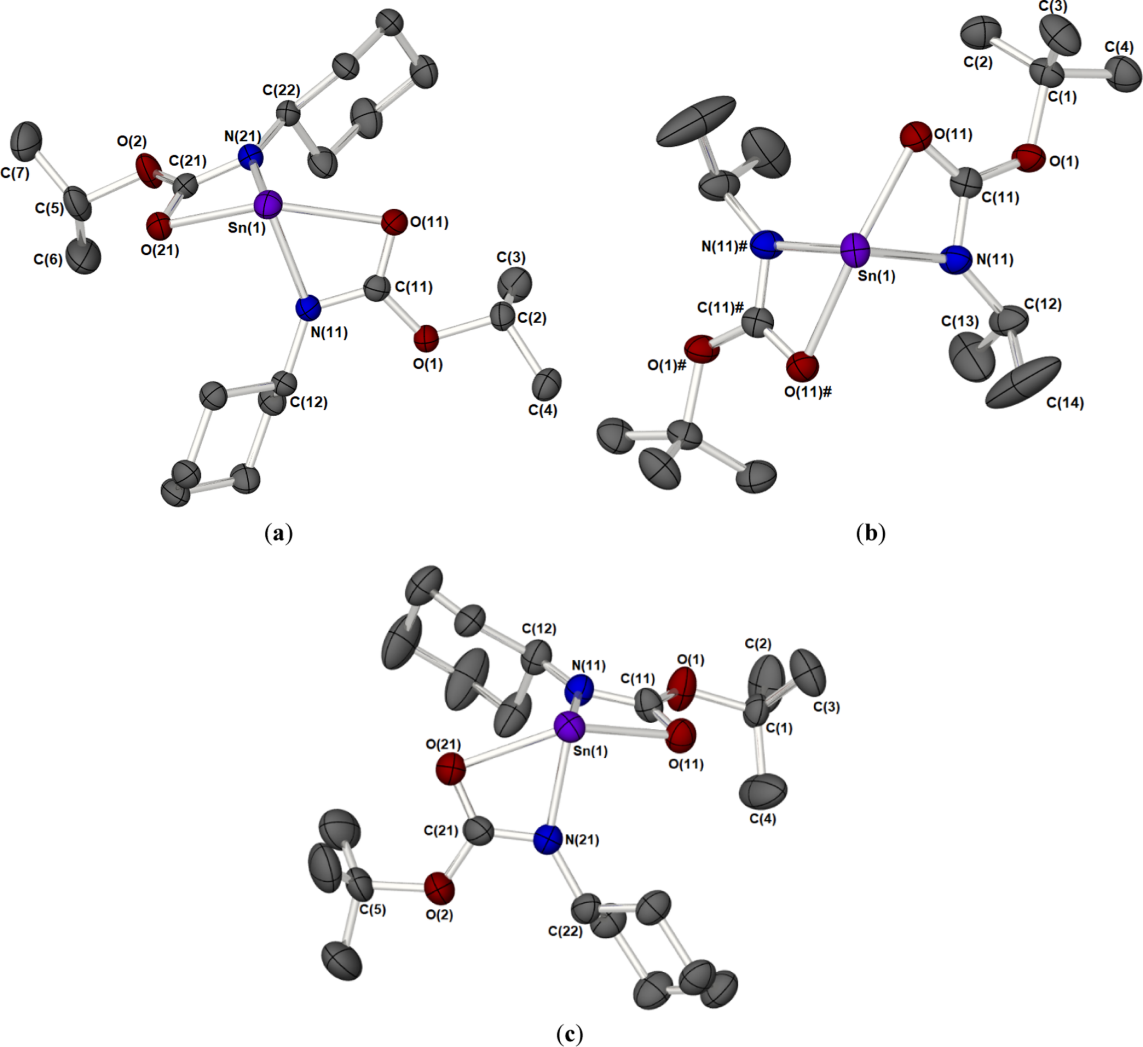
Molecular structures of compounds **6** (a), **7** (b), and **8** (c) showing the labeling of the asymmetric unit; ellipsoids are shown at 50% probability, and hydrogen atoms are omitted for clarity. Symmetry transformations are used to generate equivalent atoms **7**(**#**); 1 – *X*, *Y*, and 1/2 – *Z*.

The solid-state structures for compounds **6**–**8** were confirmed crystallographically (Figure 3). Table 3 contains selected bond lengths and angles.

**Table 3:** Selected Bond Lengths and Bond Angles for Compounds **6**–**8**

selected bond lengths (Å)			
	**6**	**7**	**8**
Sn(1)–O(11)	2.4285(15)	2.3752(18)	2.376(2)
Sn(1)–O(21)	2.3717(15)		2.399(2)
Sn(1)–N(11)	2.1703(17)	2.1723(18)	2.160(3)
Sn(1)–N(21)	2.1708(18)		2.180(3)
N(11)–C(11)	1.312(3)	1.317(3)	1.311(4)
N(11)–C(12)	1.467(3)	1.469(3)	1.462(4)
O(11)–C(11)	1.267(2)	1.268(3)	1.262(4)
O(1)–C(11)	1.347(2)	1.338(3)	1.337(4)
O(1)–C(1)	1.459(2)	1.477(3)	1.474(4)

selected bond angles (deg)			
	**6**	**7**	**8**
N(11)–Sn(1)–N(21)^a^	94.44(7)	94.54(10)	94.26(10)
N(11)–Sn(1)–O(11)	57.47(6)	58.06(7)	58.19(8)
N(11)–Sn(1)–O(21)^a^	87.50(6)	87.95(8)	86.30(9)
N(21)–Sn(1)–O(11)^a^	86.61(6)	87.95(8)	87.98(9)
N(21)–Sn(1)–O(21)^a^	58.37(6)		57.45(9)
O(11)–Sn(1)–O(21)	129.22(6)	130.82(8)	129.33(9)
C(11)–N(11)–Sn(1)	96.90(13)	95.89(14)	95.74(19)
C(11)–O(11)–Sn(1)	86.38(12)	88.01(13)	87.25(18)
O(1)–C(11)–O(11)	122.29(18)	123.8(2)	123.0(3)
C(1)–O(1)–C(11)	118.20(16)	121.42(18)	123.2(2)
C(12)–N(11)–C(11)	122.60(17)	124.2(2)	123.4(3)

a N(21) = N(11)#1, O(21) = O(11)#1 for compound **7**.

In the case of **7**, the tin atom lies on a 2-fold axis such that the asymmetric unit comprises only the metal and one ligand. All three compounds were found to exist as monomeric compounds with the tin(II) centers possessing a four-coordinate geometry in which the metals are bound to two κ^2^-*N,O* iso-carbamate ligands bound through the amido-N and carbonyl oxygen atoms of the ligand. The geometry of the complexes is heavily influenced by the stereochemically active lone pair at the tin(II) center, possessing a distorted trigonal bipyramidal geometry (disphenoidal), τ = 0.58 (**6**), 0.6 (**7**), and 0.58 (**8**), with the lone pair situated in one of the equatorial positions alongside the two nitrogen atoms of the iso-carbamate ligands. The apical positions of the bipyramid are occupied by the corresponding O-atoms of the iso-carbamate ligands. The geometric distortion observed is comparable to that seen for Sn(II) ureate systems described previously.^13e^

The variations between the three different compounds are, however, much less pronounced than those observed for dimeric systems **1**–**4**, as the amido-R group is no longer subject to steric congestion posed by the bridging alkoxide group, while the influence of the second amido substituent is mitigated by its *trans* disposition on the opposite corners of the basal plane of the square-based pyramid. Furthermore, both the Sn–O [2.3717(15)–2.4285(15) Å] and Sn–N bonds [2.160(3)–2.180(3) Å] are shorter than the corresponding bonds in **1**–**4** [2.5074(11)–2.613(1) and 2.1839(15)–2.2035(13) Å, respectively].

For compounds **6**–**8**, the {OR} group lies approximately coplanar to the NCO core of the iso-carbamate ligand, as indicated by the torsion angle {O(1)–C(11)–N(11)–C(12)}, **6**: 1.6° and 4.4°; **7**: 1.3°; **8**: 0.3 and 1.8°, which allows orbital overlap and delocalization of electron density across the backbone of the ligand system to take place. This is reflected in the bond lengths observed for C(11)–O(1) [1.337(4)–1.347(2) Å], which are ca. 0.1 Å shorter than the adjacent O(1)–C(1) bonds [1.459(2)–1.477(3) Å].

### Reversible Reaction between *^t^*Bu-NCO and Tin(II) Alkoxides

**Scheme 3:**
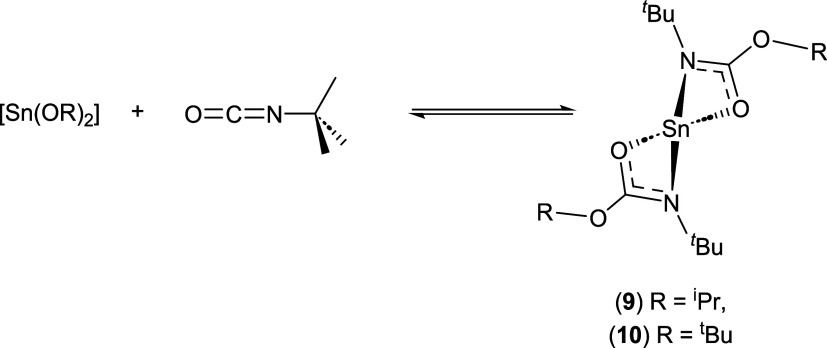
Reaction of the Sn(II) Alkoxides [Sn(O^*i*^Pr)_2_] and [Sn(O^*t*^Bu)_2_] with ^*t*^Bu-isocyanate

During the course of our investigation, several attempts were made to insert ^*t*^Bu-isocyanate into both [Sn(O^*i*^Pr)_2_] and [Sn(O^*t*^Bu)_2_] (Scheme 3).

Initial attempts to isolate insertion products **9** and **10** were unsuccessful, with the parent alkoxide being recovered from the workup and no evidence of the insertion of ^*t*^Bu-isocyanate. In the case of the isopropoxide system, in situ monitoring of the reaction in C_6_D_6_ using multinuclear NMR spectroscopy provided evidence that insertion occurred, with resonances associated with the alkoxides apparent in the 1H NMR spectra. The presence of unreacted ^*t*^Bu-isocyanate was also detected with the observation of a singlet resonance at δ 0.92 ppm.

**Figure 4:**
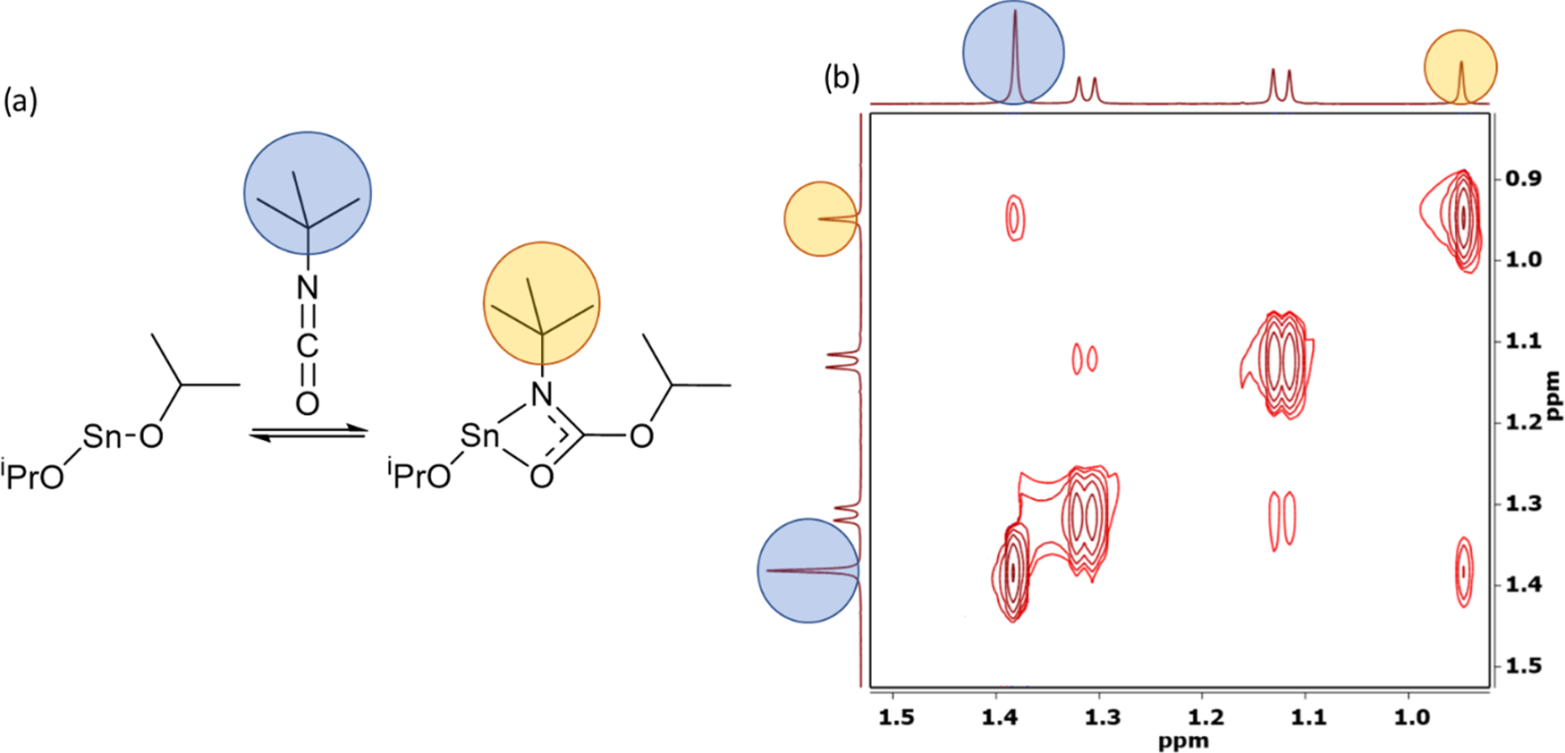
(a) Proposed reaction between [Sn(O^*i*^Pr)_2_] and ^*t*^Bu-isocyanate. (b) 2D EXSY NMR spectrum (at 298 K in *d*_8_-Tol) of the reaction between [Sn(O^*i*^Pr)_2_] and ^*t*^Bu-isocyanate showing the presence of both free ^*t*^Bu-isocyanate (blue) and ^*t*^Bu-iso-carbamate (orange).

In order to ascertain the degree of reactivity, the reactions were repeated on an NMR scale in a 1:1 ratio. The resulting ^1^H NMR spectra clearly showed a mixture of free and inserted ^*t*^Bu-isocyanate to be present in the reaction solutions, suggestive of incomplete insertion reactions in both cases and formation of the mono iso-carbamate complex [Sn{OC(N^*t*^Bu)O^*i*^Pr}{O^*i*^Pr}]. To verify this hypothesis, 2D ^1^H–^1^H exchange spectroscopy (EXSY) NMR (in *d*_8_-Tol) experiments were undertaken. 2D NMR spectra of the reaction between [Sn(O^*i*^Pr)_2_] and ^*t*^Bu-NCO clearly show (Figure 4) exchange between the two different {^*t*^Bu} resonances (i.e., inserted and noninserted) and the two different {^*i*^Pr} groups, confirming a dynamic exchange process (see the Supporting Information).

The reaction between ^*t*^Bu-NCO and [Sn(O^*t*^Bu)_2_] was slow on the NMR time scale, with broad, low intensity resonances observed in the ^1^H NMR spectra, associated with the formation iso-carbamate product, thus preventing a more detailed analysis.

These observations are entirely consistent with those made by Fulton et al. concerning the insertion of the heteroallene CO_2_ into Sn–alkoxide bonds of the BDI complexes [{BDI}SnOR] (R = ^*i*^Pr or ^*t*^Bu): the isopropoxide complex reacts both faster with the heteroallene than the corresponding *tert*-butoxide system and with a greater equilibrium constant than the corresponding *tert*-butoxide reaction.

The rate of exchange, *k*, between the inserted and free isocyanate can be readily determined by plotting the intensity of the {^*t*^Bu} resonance of the inserted iso-carbamate group (0.97 ppm), normalized against the intensity of the free isocyanate (1.40 ppm) against the mixing time.

Normalized intensities and mixing times for experiments run at 320, 326, 332, 338, and 343 K are shown in the Supporting Information, alongside experimentally determined equilibrium constants at each temperature. Below 320 K, insertion of ^*t*^Bu-isocyanate into the {Sn–O^*i*^Pr} bond is slow and results in low normalized intensities and longer mixing times. Interestingly, at temperatures above 343 K, a third {^*t*^Bu} resonance can be observed growing into the ^1^H NMR spectra at ∼1.11 ppm, which we attribute to the formation of the bis-insertion product,

Experiments across the temperature range of 320–343 K allowed us to obtain the activation parameters for this reaction. Thus, an activation energy (*E*_a_) value of 92.2 ± 0.8 kJ mol^–1^ (22.0 ± 0.2 kcal mol^–1^) was obtained from the Arrhenius plot (Figure S3), and enthalpy (Δ*H*^‡^) and entropy (Δ*S*^‡^) of activation values of 89.4 ± 0.8 kJ mol^–1^ (21.3 ± 0.2 kcal mol^–1^) and 12.6 ± 2.9 J mol^–1^ K^–1^ (3.0 ± 0.7 kcal mol^–1^ K^–1^), respectively, were obtained from the Eyring plot (Figure S4). While the enthalpy value is consistent with a slow insertion process, the entropy value is large and positive, which indicates a dissociative pathway and a highly ordered transition state in the rate-determining step of the reaction. Such observations could be consistent with a reaction in which the oligomeric Sn(II)–alkoxide species would require initial deaggregation in order for the insertion reaction to occur.

### Reversible Reaction between CO_2_ and Tin(II) Alkoxides

The steric and electronic nature of both alkoxide groups and electrophilic alkyl/aryl isocyanates clearly plays a significant role in the reactivity and reversibility of these reactions. As noted previously, Sn(II) alkoxides are considered to be relatively weak nucleophiles, and CO_2_ is more electrophilic than alkyl or aryl isocyanates. Fulton et al. previously showed the insertion of CO_2_ into the Sn–alkoxide bonds of the BDI complexes [{BDI}SnOR] (R = ^*i*^Pr, ^*s*^Bu, ^*t*^Bu) to be both temperature-dependent and reversible;^9b^ to the best of our knowledge, the insertion of CO_2_ into either [Sn(O^*i*^Pr)_2_] or [Sn(O^*t*^Bu)_2_] to produce Sn(II) carbonate systems (Figure 1) has not previously been explored.

Unfortunately, the direct reaction of [Sn(O^*i*^Pr)_2_] or [Sn(O^*t*^Bu)_2_] with CO_2_ to prepare tin(II) carbonates proved unsuccessful. For example, when [Sn(O^*t*^Bu)_2_] was stirred under an atmosphere of CO_2_, a white precipitate was formed on cooling to −30 °C. However, this product decomposed with evolution of gas on removal from the CO_2_ atmosphere. ^119^Sn{^1^H} NMR spectroscopy of the reaction mixture under a positive pressure of CO_2_ showed the presence of a single resonance at −91 ppm, consistent with [Sn(O^*t*^Bu)_2_].^14^ Although a similar reaction with [Sn(O^*i*^Pr)_2_] also afforded only starting alkoxide on workup, monitoring the reaction of ^13^CO_2_, in situ, by NMR spectroscopy confirmed the formation of the desired {SnO_2_COR} species via the observation of a ^13^C resonance at ca. 160 ppm, characteristic of the C=O moiety.

To allow a more comprehensive analysis of the formation of tin(II) carbonate complexes, the reactions of [Sn(O^*i*^Pr)_2_] or [Sn(O^*t*^Bu)_2_] with CO_2_ were carried out in sealed Youngs tap NMR tubes, thus allowing the carbonate compounds to be analyzed in situ using multinuclear NMR spectroscopy. The formation of tin(II) carbonate species can be most clearly observed in the ^13^C NMR spectra through the appearance of a characteristic resonance observed at δ 155–162 ppm corresponding to the quaternary carbon bound to three oxygen atoms.

The reaction of [Sn(O^*i*^Pr)_2_] with ^13^CO_2_ was monitored by multinuclear NMR spectroscopy. The sample was pressurized with ^13^CO_2_ until free ^13^CO_2_ was detected in the ^13^C{^1^H} NMR spectrum, i.e., δ 125 ppm in *d*_8_-Tol. Analysis of the ^13^C{^1^H} NMR spectrum showed not only the presence of the carbonate carbon at 161.6 ppm but also two different environments for the alkyl substituents, with resonances observed at δ 70.4 and 67.1 ppm for the {^*i*^Pr} methine carbons and δ 28.1 and 22.7 ppm for the methyl carbons. The presence of two environments was confirmed in the ^1^H NMR spectrum, which displayed two doublet resonances at δ 1.15–1.19 and 1.23–1.27 ppm (methyl groups) and two multiplet signals at δ 4.51 and 4.85 ppm (methine groups). As free CO_2_ was detected in the ^13^C{^1^H} NMR spectrum, the sample was judged to be saturated with CO_2_ with the establishment of an equilibrium between the stannous carbonate and parent alkoxide. Additionally, ^119^Sn{^1^H} NMR spectroscopy showed a single peak in the spectrum at −368 ppm (cf. [Sn(O^*i*^Pr)_2_] δ = −211 ppm (*d*_8_-Tol)).^14^

**Figure 5:**
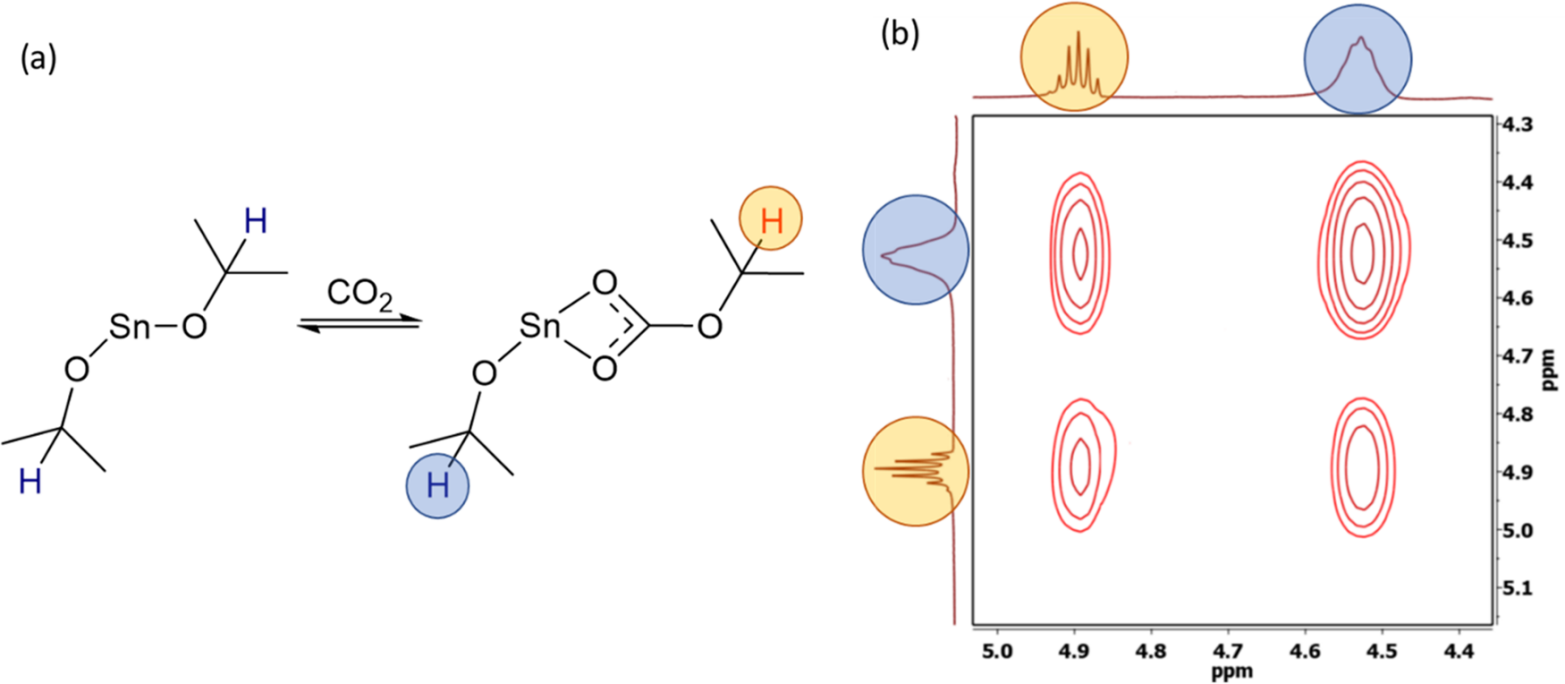
(a) Proposed reaction between [Sn(O^*i*^Pr)_2_] and CO_2_. (b) 2D EXSY NMR spectrum (at 268 K in *d*_8_-Tol) of the reaction between [Sn(O^*i*^Pr)_2_] and CO_2_, showing the methine protons of both the isopropoxide group,{Sn-OCHMe_2_}, (blue) and the isopropoxy carbonate group, {Sn–O_2_COCHMe_2_}, (orange), showing exchange.

To investigate this apparent equilibrium process, a 2D EXSY NMR experiment was conducted, which showed that the resonances for the carbonate and alkoxide were in exchange on an NMR time scale (Figure 5). This confirmed the reversibility and highlighted the highly facile nature of the reaction.

Utilizing 1D EXSY NMR experiments (in *d*_8_-Tol), it was possible to obtain kinetic data for the equilibrium between the tin(II) carbonate and the parent alkoxide [Sn(O^*i*^Pr)_2_] between 266 and 303 K. Full experimental detail including values calculated for the rate constant, K, are shown alongside the temperatures for the individual experiments in the Supporting Information.

The equilibrium between [Sn(O^*i*^Pr)_2_] and the metallo-alkyl carbonate, [Sn{O_2_CO^*i*^Pr}{O^*i*^Pr}], was measured at six different temperatures, and Δ*H*^0^ (68.0 ± 1.3 kJ mol^–1^ or 16.3 ± 0.3 kcal mol^–1^) and Δ*S*^0^ (−8.7 ± 2.8 J mol^–1^ K^–1^ or −2.1 ± 0.7 kcal mol^–1^ K^–1^) were calculated for the insertion of CO_2_ into the Sn–O*^i^*Pr bond alongside an activation energy (*E*_a_) value for the reaction of 70.3 ± 1.3 kJ mol^–1^ (16.8 ± 0.3 kcal mol^–1^).

Attempts to run comparable 2D EXSY experiments for the reaction between [Sn(O^*t*^Bu)_2_] and CO_2_ were unsuccessful due to the resonances in the ^1^H NMR attributed to the carbonate and the alkoxide possessing similar chemical shifts. The ^1^H NMR and ^13^C{^1^H} NMR spectra did, however, evidence the presence of both alkoxide and carbonate peaks. For the reaction of [Sn(O^*t*^Bu)_2_] with CO_2_, the ^1^H NMR spectrum comprised resonances at δ 1.27 and 1.42 ppm. The corresponding ^13^C{^1^H} NMR spectrum had resonances at δ 34.7 and 33.6 ppm associated with the methyl groups, resonances at δ 73.6 and 74.6 were associated with the quaternary {^*t*^Bu} carbon atoms, and resonances at 157.9 ppm were associated with the carbonate carbon atom.

## Conclusions

We have shown that simple tin(II) alkoxides [Sn(O^*i*^Pr)_2_] and [Sn(O^*t*^Bu)_2_] are capable of reacting with heteroallene electrophiles such as isocyanates (R-NCO) to form a series of mono iso-carbamate complexes, of the general form [Sn{κ^2^-*N,O*-R′-NC(OR)O}(μ-OR)]_2_, (R = ^*i*^Pr or ^*t*^Bu), when the {R′} group is either R′ = 2,4,6-trimethylphenyl (Mes) or 2,6-diisopropylphenyl (Dipp), and bis-iso-carbamate complexes, of the general form [Sn{κ^2^-*N,O*-R′-NC(O^*i*^Pr)O}_2_] (R = ^*i*^Pr or ^*t*^Bu) when the R′ group is either isopropyl (^*i*^Pr) or cyclohexyl (Cy). In the case of the mono iso-carbamate products, solution-state multinuclear NMR spectroscopy clearly indicates a complicated ligand exchange process in which the mono-insertion product isolated in the solid-state undergoes deinsertion of the isocyanate in solution. While speculative assessment of ^1^H DOSY experiments can be made, they are inconclusive, and attempts to isolate products other than mono iso-carbamate products **1**–**4** were unsuccessful. Contrastingly, the reaction of [Sn(O^*i*^Pr)_2_] and [Sn(O^*t*^Bu)_2_] with either ^*i*^Pr-CNO or Cy-NCO showed exclusive formation of bis-insertion products **5**–**8**.

^1^H NMR studies show that the reaction of ^*t*^Bu-NCO with either [Sn(O^*i*^Pr)_2_] or [Sn(O^*t*^Bu)_2_] results in a reversible mono-insertion. Variable-temperature 2D ^1^H–^1^H exchange spectroscopy (VT-2D-EXSY) was thus used to determine the rate of exchange between free ^*t*^Bu-NCO and the coordinated ^*t*^Bu-iso-carbamate ligand for the {O^*i*^Pr} alkoxide complex, as well as the activation energy (*E*_a_ = 92.2 ± 0.8 kJ mol^–1^ or 22.0 ± 0.2 kcal mol^–1^), enthalpy (Δ*H*^‡^ = 89.4 ± 0.8 kJ mol^–1^ or 21.3 ± 0.2 kcal mol^–1^), and entropy (Δ*S*^‡^ = 12.6 ± 2.9 J mol^–1^ K^–1^ or 3.0 ± 0.7 kcal mol^–1^ K^–1^) for the process [Sn(O^*i*^Pr)_2_] + ^*t*^Bu-NCO ↔ [Sn{κ^2^-N,O-^*t*^Bu-NC(O^*i*^Pr)O}(O^*i*^Pr)].

As part of our study, attempts to form Sn(II) alkyl carbonates by the insertion of ^13^CO_2_ into either [Sn(O^*i*^Pr)_2_] or [Sn(O^*t*^Bu)_2_] proved unsuccessful. However, VT-2D-EXSY (^1^H) confirmed the reversible alkyl carbonate formation (*E*_a_ = 70.3 ± 1.3 kJ mol^–1^ or 16.8 ± 0.3 kcal mol^–1^; Δ*H*^‡^ = 68.0 ± 1.3 kJ mol^–1^ or 16.3 ± 0.3 kcal mol^–1^ and Δ*S*^‡^ = −8.1 ± 2.8 J mol^–1^ K^–1^ or −2.1 ± 0.7 kcal mol^–1^ K^–1^).

## Experimental Section

All reactions dealt with potential air- and moisture-sensitive compounds, and, as such, were carried out under an argon atmosphere using standard Schlenk line and glovebox techniques in an MBraun Labmaster glovebox at O_2_, H_2_O < 1.5 ppm. NMR experiments were conducted in Youngs tap NMR tubes prepared and sealed in a glovebox under argon and were recorded on a Bruker AV-300 or a 500 MHz Bruker Avance II+. The spectra were referenced relative to residual solvent resonances. Unless otherwise stated, data quoted were recorded at 298 K. For complexes **3** and **4**, NMR data reported in the Experimental Section (^1^H, ^13^C{^1^H}, and ^119^Sn{1H}) were recorded in *d*_8_-Tol at 373 K. Elemental analysis was performed at SACS, London Metropolitan University. Solvents for air- and moisture-sensitive reactions were provided by an Innovative Technology Solvent Purification System or dried/degassed manually according to established laboratory procedures. [Sn(O^*i*^Pr)_2_] and [Sn(O^*t*^Bu)_2_] were produced according to literature procedures.^14,15^ All other reagents were purchased from commercial sources and used without further purification. No uncommon hazards are noted.

### Synthesis of [Sn_2_{μ-O^*i*^Pr}_2_{κ^2^-*N*,*O*-Mes-NC(O^*i*^Pr)O}_2_] (**1**)

A toluene solution (5 mL) of mesityl isocyanate (322 mg, 2 mmol) was added dropwise, over 5 min, to a stirred toluene solution (15 mL) of [Sn(O^*i*^Pr)_2_] (237 mg, 1 mmol). After the mixture was stirred at room temperature for 1 h, toluene was removed in vacuo. To the residual solid, 5 mL of fresh toluene was added, and the solid was heated into solution. After filtration through Celite, the solution was stored at 0 °C for 3 days, during which colorless crystalline blocks were formed. The product was subsequently isolated by filtration and dried in vacuo. Yield: 394 mg, 99%. Analysis found (%) (calcd for C_16_H_25_N_1_O_3_Sn): C 48.46 (48.27), H 6.43 (6.33), N 3.37 (3.52). ^1^H NMR (500 MHz, C_6_D_6_); δ 1.02 (d, *J*_H–H_ = 5.0 Hz, 6H, {COCH*Me*_2_}), 1.07 (m, 6H, {SnOCH*Me*_2_}), 2.12 (s, 3H, {*p*-C*H*_3_}), 2.50 (s, 6H, {*o*-C*H*_3_}), 4.34 (sept, ^3^*J*_H–H_ = 5.0 Hz 1H, {SnOC*H*Me_2_}), 5.08 (sept, ^3^*J*_H–H_ = 5.0 Hz, 1H, {COC*H*Me_2_}), 6.82 (s, 2H, {*m*-CH}). ^13^C (126 MHz, C_6_D_6_) δ 19.9 ({*o*-*C*H_3_}), 20.9 ({*p*-*C*H_3_}), 22.4 ({COCH*Me*_2_}), 25.6 ({SnOCHMe*Me*}), 26.3 ({SnOCHMe*Me*}), 68.8 ({CO*C*HMe_2_} and {SnO*C*HMe_2_}), 129.0 ({*m*-*C*H}), 134.0 ({*p*-*C*Me}), 134.4 ({*o*-*C*Me}), 139.4 ({*C*–N}), 161.7 ({N*C*(O)O^i^Pr}). ^119^Sn{^1^H} NMR (186 MHz, C_6_D_6_) δ −313.

### Synthesis of [Sn_2_{μ-O^*i*^Pr}_2_{κ^2^-*N*,*O*-Dipp-NC(O^*i*^Pr)O}_2_] (**2**)

Following the same procedure for the synthesis of **1**, a toluene solution (15 mL) of 2,6-diisopropylphenyl isocyanate (406 mg, 2 mmol) was added dropwise, over 5 min, to a stirred toluene solution (15 mL) of [Sn(O^*i*^Pr)_2_] (237 mg, 1 mmol). After stirring at room temperature for 1 h, toluene was removed in vacuo. To the residual solid, 5 mL of fresh toluene was added, and the solid was heated into solution. After filtration through Celite, the solution was stored at 0 °C for 3 days, during which colorless crystalline blocks were formed. The product was subsequently isolated by filtration and dried in vacuo. Yield: 423 mg, 96%. Analysis found (%) (calcd for C_19_H_31_N_1_O_3_Sn): C 51.36 (51.85), H 7.18 (7.10), N 3.17 (3.18). ^1^H NMR (500 MHz, C_6_D_6_); δ 1.03 (d, ^3^*J*_H–H_ = 6.3 Hz, 6H, {COCH*Me*_2_}), 1.10 (d, ^3^*J*_H–H_ = 5.4 Hz, 6H, {SnOCH*Me*_2_}), 1.34 (d, ^3^*J*_H–H_ = 6.7 Hz, 6H, {C–CH*Me*Me}) 1.38 (d, ^3^*J*_H–H_ = 6.8 Hz, 6H, {C–CHMe*Me*}) 3.69 (m, 2H, {C–C*H*Me_2_}), 4.30 (m, 1H, {SnOC*H*Me_2_}), 5.07 (m, 1H, {C–C*H*Me_2_}) 7.03 (s, 1H, {*p*-CH}), 7.08 (s, 2H, {*m*-CH}). ^13^C (126 MHz, C_6_D_6_) δ 22.3 ({COCH*Me*_2_}), 24.3 ({SnOCH*Me*Me}), 25.4 ({SnOCHMe*Me*}) 25.9 ({C–CHMe*Me*}). 26.5 ({C–CH*Me*Me}), 29.1 ({C–*C*HMe_2_}), 68.8 ({SnO*C*HMe_2_}), 69.4 ({CO*C*HMe_2_}), 132.4 ({*m*-*C*H}), 126.1 ({*p*-*C*Me}), 138.9 ({*o*-*C*^i^Pr}), 145.2 ({*C*–N}), 162.0 ({N*C*(O)O^i^Pr}). ^119^Sn{^1^H} NMR (186 MHz, C_6_D_6_) δ −345.

### Synthesis of [Sn_2_{μ-O^*t*^Bu}_2_{κ^2^-*N*,*O*-Mes-NC(O^*t*^Bu)O}_2_] (**3**)

Following the same procedure for the synthesis of **1**, mesityl isocyanate (322 mg, 2 mmol in 15 mL) was added to a toluene solution (15 mL) of [Sn(O^*t*^Bu)_2_](265 mg, 1 mmol). Subsequent extraction and filtration, followed by storage at −5 °C, resulted in the formation of colorless crystals, which were isolated by filtration, washed with cold hexanes, and dried in vacuo. Yield: 414 mg, 97%. Analysis found (%) (calcd for C_18_H_29_N_1_O_3_Sn): C 50.68 (50.73), H 6.89 (6.86), N 3.29 (3.29). ^1^H NMR (500 MHz, *d*_8_-Tol, 373 K); δ 1.35 (br s, 9H, {COC*Me*_3_}), 1.41 (br s, 9H, {SnOC*Me*_3_}), 2.01 (s, 3H, {*p*-C*H*_3_}) 2.02 (br s, 6H, {*p*-C*H*_3_}) 6.54 (s, 2H, {*m*-CH}). ^13^C (126 MHz, *d*_8_-Tol, 373 K) δ 18.3 ({*o*-C*H*_3_}), 29.0 ({*p*-C*H*_3_}), 34.2 ({COC*Me*_3_}) 34.92 ({SnOC*Me*_3_}). 75.0 ({SnO-*C*Me_3_}), 84.3 ({SnO*C*Me_3_}), 132.9 ({*o*-*C*Me}), 135.1 ({*p*-*C*Me}), 137.7 ({*m*-*C*H}), 141.0 ({*C*–N}), and 161.4 ({N*C*(O)O^*t*^Bu}). ^119^Sn{^1^H} NMR (186 MHz, *d*_8_-Tol, 373 K) δ: −104, −107, and −281.

### Synthesis of [Sn_2_{μ-O^*t*^Bu}_2_{κ^2^-*N*,*O*–Dipp-NC(O^*t*^Bu)O}_2_] (**4**)

Following the same procedure for the synthesis of **1**, 2,6-diisopropylphenyl isocyanate (406 mg, 2 mmol) was added to a toluene solution (15 mL) of [Sn(O^*t*^Bu)_2_] (265 mg, 1 mmol). Subsequent extraction and filtration, followed by storage at −5 °C, resulted in the formation of colorless crystals, which were isolated by filtration, washed with cold hexanes, and dried in vacuo. Yield: 440 mg, 94%. Analysis found (%) (calcd for C_21_H_35_N_1_O_3_Sn): C 53.86 (53.87), H 7.58 (7.53), N 3.05 (2.99). ^1^H NMR (500 MHz, *d*_8_-Tol, 373 K); δ 1.08 (d, *J*_H–H_ = 6.8 Hz, 6H, {C–CH*Me*_2_}), 1.38–1.42 (br m, 18H, {SnOC*Me*_3_} & {COC*Me*_3_}), 3.11 (d, ^3^*J*_H–H_ = 6.8 Hz, 6H, {C–*C*HMe_2_}) 6.94–6.97 (m, 2H, {*m*-CH}), 7.05 (m, 1H, {*p*-CH}). ^13^C (126 MHz, *d*_8_-Tol, 373 K) δ 22.9 ({CCH*Me*_2_}), 24.3 ({SnOC*Me*_3_}), 30.2 ({C*C*HMe_2_}) 34.9 ({C–OC*Me*_3_}), 74.2 ({Sn–O*C*Me_3_}), 84.0 ({C–O*C*Me_3_}), 123.9 ({*p*-*C*H}), 126.5 ({*o*-*C*^*i*^Pr}), 137.7 ({*m*-*C*H}), 143.6 ({*C*–N}), 163.5 ({N*C*(O)O^i^Pr}). ^119^Sn{^1^H} NMR (186 MHz, *d*_8_-Tol, 373 K) δ −104 and −141.

### Synthesis of [Sn{κ^2^-*N*,*O*-^i^Pr-NC(O^i^Pr)O}_2_] (**5**)

To a solution of [Sn(O^*i*^Pr)_2_] (400 mg, 1.68 mmol) in toluene (5 mL), isopropyl isocyanate (287 mg, 3.36 mmol) was added. The solution was vigorously stirred. Storage of the reaction mixture at 4 °C resulted in the formation of a white crystalline material, which was isolated by filtration and dried in vacuo. Yield: 672 mg, 98%. Analysis found (calcd for C_14_H_28_N_2_O_4_Sn): C 41.1 (41.3), H 7.04 (6.93), N 6.74 (6.88)%. ^1^H NMR (300 MHz, *d*_8_-Tol) δ 1.13 (d, ^3^*J*_H–H_ = 6.0 Hz, 6H, OCH*Me*_2_), 1.19 (d, ^3^*J*_H–H_ = 6.4 Hz, 6H, NCH*Me*_2_), 4.04 (sept, ^3^*J*_H–H_ = 6.4 Hz, 1H, NC*H*Me_2_), 4.94 (sept, ^3^*J*_H–H_ = 6.0 Hz, 1H, OC*H*Me_2_). ^13^C NMR (74 MHz, *d*_8_-Tol); δ 22.9 (OCH*Me*_2_), 24.3 (NCH*Me*_2_), 45.9 (N*C*HMe2), 69.6 (O*C*HMe_2_), 163.2 ({N*C*(O)O^i^Pr}). ^119^Sn NMR (112 MHz, *d*_8_-Tol); δ −360 ppm.

### Synthesis of [Sn{κ^2^-*N*,*O*-Cy-NC(O^i^Pr)O}_2_] (**6**)

Cyclohexyl isocyanate (431 μL, 3.38 mmol) and [Sn(O^*i*^Pr)_2_] (400 mg, 1.68 mmol) were added to toluene (5 mL). The solution was concentrated in vacuo to give a yellow microcrystalline material. Removal of the solvent in vacuo followed by recrystallization from hexanes at −30 °C resulted in crystals suitable for X-ray diffraction analysis. Yield 723 mg, 89%. Analysis found (calcd for C_20_H_36_N_2_O_4_Sn): C 50.2 (49.30), H 7.75 (7.45), N 5.74 (5.75)%. ^1^H NMR (300 MHz, d^8^ tol) δ ppm 1.10–1.28 (m, 10H, CH_3_, CH_2_), 1.44–1.57 (m, 4H, CH_2_), 1.67–1.71 (m, 2H, CH_2_), 3.64–3.74 (m, 1H, NCH), 4.97 (sept, ^3^*J*_H–H_ = 6.2 Hz 1H, OCH*Me*_2_). ^13^C NMR (74 MHz, *d*^8^-Tol); δ ppm 22.9 (OCH*Me*_2_), 26.5(*C*H_2_), 26.7 (*C*H_2_), 34.8 (*C*H_2_), 53.9 (N*C*H), 69.6 (O*C*HMe_2_), 163.2 ({N*C*(O)O^i^Pr}). ^119^Sn NMR (112 MHz, *d*^8^-Tol); δ ppm −355 ppm.

### Synthesis of [Sn{κ^2^-*N*,*O*-^*i*^Pr-NC(O^t^Bu)O}_2_] (**7**)

Isopropyl isocyanate (266 mg, 3.12 mmol) and [Sn(O^*t*^Bu)_2_] (400 mg, 1.56 mmol) were added to toluene (5 mL). The solution was concentrated in vacuo to give a yellow microcrystalline material. Removal of the solvent in vacuo followed by recrystallization from hexanes at −30 °C resulted in crystals suitable for X-ray diffraction analysis. Yield 462 mg, 68%. Analysis found (calcd for C_16_H_32_N_2_O_4_Sn): C 44.20 (44.16), H 7.45 (7.41), N 6.74 (6.44)%. ^1^H NMR (300 MHz, *d*_8_-Tol) δ ppm 1.20 (d, ^3^*J*_H–H_ = 6.4 Hz, 6H, CH*Me*_2_), 1.43 (s, 9H, C*Me*_3_), 3.95 (sept, ^3^*J*_H–H_ = 6.4 Hz, 1H, *C*HMe_2_). ^13^C NMR (74 MHz, *d*_8_-Tol); δ ppm 24.2 (CHMe_2_), 29.3 (C*Me*_3_), 49.1 (*C*HMe_2_), 80.3 (*C*Me_3_), 163.2 ({N*C*(O)O^*t*^Bu}). ^119^Sn NMR (112 MHz, *d*_8_-Tol); δ ppm −338 ppm

### Synthesis of [Sn{κ^2^-*N*,*O*-Cy-NC(O^*t*^Bu)O}_2_] (**8**)

Cyclohexyl isocyanate (399 μL, 3.12 mmol) and [Sn(O^*t*^Bu)_2_] (400 mg, 1.56 mmol) were added to hexanes (5 mL). The solution was concentrated in vacuo to give a yellow microcrystalline material. Removal of the solvent in vacuo followed by recrystallization from hexanes at −30 °C resulted in crystals suitable for X-ray diffraction analysis. Yield 588 mg, 73%. Analysis found (calcd for C_22_H_40_N_2_O_4_Sn): C 51.1 (51.3), H 7.85 (7.82), N 5.52 (5.44)%. ^1^H NMR (300 MHz, *d*_8_-Tol); δ 1.44 (s, 9H, C*Me*_3_), 1.50–1.75 (m, 8H, C*H*_2_), 1.80–1.91 (m, 2H, C*H*_2_), 3.57–3.76 (m, 1H, C*H*). ^13^C NMR (74 MHz, *d*_8_-Tol); δ 26.5 (C*Me*_3_), 26.6 (*C*H_2_), 29.3 (*C*H_2_), 34.6 (*C*H_2_), 54.2 (*C*H), 80.3 (*C*Me_3_), 163.1 ({N*C*(O)O^*t*^Bu}). ^119^Sn NMR (112 MHz, *d*_8_-Tol); δ −335 ppm.

### Single-Crystal X-ray Diffraction

Experimental details related to the single-crystal X-ray crystallographic studies for compounds **1**–**4** and **6**–**8** are summarized in the Supporting Information (Table S1). Crystallographic data were collected at 150(2) K on either a Nonius Kappa CCD diffractometer using radiation Mo–Kα (λ = 0.71073 Å) or a SuperNova, Dual, EosS2 diffractometer using radiation Cu–Kα (λ = 1.54184 Å) or Mo–Kα (λ = 0.71073 Å). All structures were solved by direct methods followed by full-matrix least-squares refinement on F2 using the WINGX-2014 suite of programs^20^ or OLEX2.^21^ All hydrogen atoms were included in idealized positions and refined using the riding model.

Crystals were isolated from an argon-filled Schlenk flask and immersed under oil before being mounted onto the diffractometer.

## Supporting Information Available

Crystallographic data for complexes **1**–**4** and **6**–**8**; experimental details for ^1^H–^1^H exchange spectroscopy of the 1:1 reaction of *^t^*Bu-NCO with [Sn{O*^i^*Pr}_2_] at 320.4, 326.1, 331.8, 337.5, and 343.2 K and of [Sn{O^i^Pr}_2_] with CO_2_ at 266.6, 271.9, 277.4, 288.0, 297.9, and 302.8 K, including Arrhenius and Eyring plots of the experimentally determined equilibrium rate constants at different temperatures; and selected NMR spectra for complexes **1**–**4** (^1^H, ^119^Sn, and ^1^H DOSY) (PDF)
