# Supplementary material for: Heteroallene Insertions into Tin(II) Alkoxide Bonds
Source: Inorg Chem. 2024 Jun 4;63(24):10967–79. doi: 10.1021/acs.inorgchem.3c04551 (PMC11190973; doi:10.1021/acs.inorgchem.3c04551)
Supplement: Supplementary file 1 — ic3c04551_si_001.pdf [file ic3c04551_si_001.pdf]

Heterocumulene Insertions into Tin(II) Alkoxide Bonds

Aiden Ryan,[a] Andrew Brookes,[a,b] Andrew J Straiton,[a] Thomas Wildsmith [a,b] John P. Lowe,[c] Kieran C. Molloy,[a] Michael S. Hill[a]* and Andrew L. Johnson.[a]*

a: Department of Chemistry, University of Bath, Claverton Down, Bath BA2 7AY, United Kingdom.

b: Centre for Sustainable Chemical Technologies, University of Bath, Bath BA2 7AY, United Kingdom.

c: Material and Chemical Characterisation Facility (MC²), University of Bath, United Kingdom. BA2 7AY.

Table S1: Crystallographic data for the complexes **1** to **4**.

Compound	**1**	**2**	**3**	**4**
Empirical Formula	$C_{32}H_{50}N_2O_6Sn_2$	$C_{38}H_{62}N_2O_6Sn_2$	$C_{36}H_{58}N_2O_6Sn_2$	$C_{49}H_{78}N_2O_6Sn_2$
Formula mass	796.12	880.27	852.22	1028.51
Crystal System	Triclinic	Monoclinic	Monoclinic	Triclinic
Space Group	$P\overline{1}$	$P2_1/n$	$P2_1/n$	$P\overline{1}$
Wavelength (Å)	0.71073	0.71073	0.71073	1.54184
a (Å)	8.4233(10)	19.49450(10)	10.18870(10)	13.2944(3)
b (Å)	10.9429(14)	10.26860(10)	14.4250(3)	13.3620(3)
c (Å)	11.020(4)	22.9661(2)	13.7210(3)	15.7524(3)
α (°)	69.105(5)	90	90	75.777(2)
β (°)	89.151(4)	113.9014(3)	101.8319(11)	68.446(2)
γ (°)	72.067(9)	90	90	88.816(2)
V (Å^3)	897.7(4)	4203.13(6)	1973.76(6)	2515.27(10)
ρ (mg/m^3)	1.473 Mg/m^3	1.391	1.434	1.358
Z	1	4	2	2
Crystal size (mm^3)	0.225 x 0.100 x 0.075	0.250 x 0.200 x 0.200	0.300 x 0.250 x 0.200	0.423 x 0.217 x 0.159
Reflections collected	16962	71441	35579	29582
Independent reflections	5445 [R(int) = 0.0459]	12749 [R(int) = 0.0401]	6271 [R(int) = 0.0634]	10036 [R(int) = 0.0396]
Goodness-of-fit on F^2	1.071	1.116	1.059	1.076
Data / restraints / parameters	5445 / 0 / 197	12749 / 0 / 449	6271 / 0 / 217	10036 / 0 / 551
Final R indices [I>2sigma(I)]	R_1 = 0.0266, wR_2 = 0.0628	R_1 = 0.0234, wR_2 = 0.0575	R_1 = 0.0240, wR_2 = 0.0560	R_1 = 0.0345, wR2 = 0.0904
R indices (all data)	R_1 = 0.0329, wR_2 = 0.0654	R_1 = 0.0269, wR_2 = 0.0598	R_1 = 0.0311, wR_2 = 0.0589	R_1 = 0.0353, wR_2 = 0.0914
Res. e density (eÅ^3)	0.947 and -1.129	1.132 and -0.813	0.851 and -0.942	1.222 and -2.095
CCDC number	2246543	2246544	2246545	2246546

Table S2: Crystallographic data for the complexes **6** to **8**.

Compound	**6**	**7**	**8**
Empirical Formula	$C_{20}H_{36}N_2O_4Sn$	$C_{16}H_{32}N_2O_4Sn$	$C_{22}H_{40}N_2O_4Sn$
Formula mass	487.20	435.12	515.25
Crystal System	Triclinic	Orthorhombic	Monoclinic
Space Group	$P\bar{1}$	$C222_1$	$P2_1/n$
Wavelength (Å)	0.71073	0.71073	0.71073
a (Å)	9.9867(2)	9.7150(2)	9.4881(2)
b (Å)	11.2071(4)	10.7320(2)	29.6626(6)
c (Å)	11.2475(4)	20.0140(3)	10.0492(2)
α (°)	75.7317(16)	90	90
β (°)	71.8450(17)	90	114.8535(11)
γ (°)	81.5122(18)	90	90
V (Å^3)	1155.84(6)	2086.69(7)	2566.32(9)
ρ (mg/m^3)	1.400	1.385	1.334
Z	2	4	4
Crystal size (mm^3)	0.200 x 0.200 x 0.150	0.200 x 0.170 x 0.080	0.150 x 0.125 x 0.100
Reflections collected	20292	71441	34176
Independent reflections	5280 [R(int) = 0.0444]	12749 [R(int) = 0.0401]	4960 [R(int) = 0.0451]
Goodness-of-fit on F^2	1.038	1.142	1.108
Data / restraints / parameters	5280 / 0 / 248	2809 / 0 / 112	4960 / 0 / 268
Final R indices [I>2sigma(I)]	R_1 = 0.0278, wR_2 = 0.0617	R_1 = 0.0230, wR_2 = 0.0562	R_1 = 0.0340, wR_2 = 0.0779
R indices (all data)	R_1 = 0.0366, wR_2 = 0.0649	R_1 = 0.0259, wR_2 = 0.0577	R_1 = 0.0455, wR_2 = 0.0830
Res. e density (eÅ^3)	0.489 and -0.626	1.168 and -0.771	0.638 and -0.704
CCDC number	2246547	2246548	2246549

2D 1H-1H Exchange Spectroscopy (EXSY) NMR experiments:

Utilising 1D EXSY NMR experiments it was possible to obtain kinetic data for the equilibrium between the tin(II) iso-carbamate and the parent alkoxide i.e. [Sn{O^iPr}$_2$] using a method adapted from Nikonov and co-workers.[1]

A series of SELNOPG (selective-ge 1D EXSY) NMR experiments were conducted at varying mixing time and temperature. The SELNOPG NMR selectively irradiates part of the spectra – one of the resonances observed to be in exchange – with a series of 90 ° and selective 180 ° pulses. This is followed by a second 90° pulse after a short pause, referred to as the mixing time. The initial selective irradiation causes the irradiated peak to be inverted, but as the peaks are in exchange a secondary resonance should appear and the initially irradiated resonance will decrease in size. The rate of exchange, *k*, can be determined by plotting the intensity of the new resonance (normalised against the intensity of the original peak) against the mixing time (Figure S2). In Table S7 the values calculated for *k* are shown alongside the temperature for the individual experiments.

Figure S1: ^{1}H NMR Spectrum (C_6H_6) of the 1:1 reaction of tBuNCO with [Sn{O^{i}Pr}$_2$] at 298K.

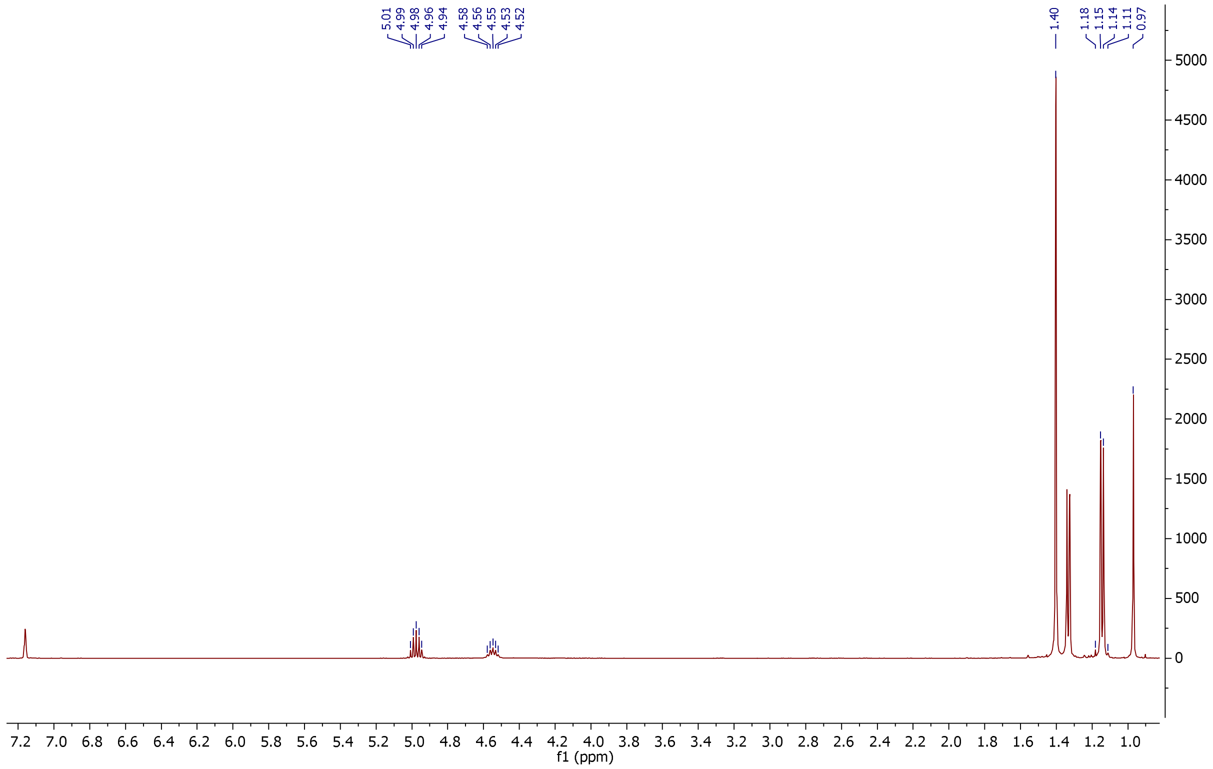

Table S3: Mixing time and normalised intensities for the reaction of [Sn{O^{i}Pr}$_2$] with ^{t}BuNCO at 320.4, 326.1, 331.8, 337.5 and 343.2K respectively.

Temp K		**343.2**	**337.5**	**331.8**	**326.1**	**320.4**
Temp oC		70.05	64.35	58.65	52.95	47.25
		Normalized Intensities				
Mixing Time (s)						
**0.01**		0.009991	0.005865	0.003317	0.001793	0.000856
**0.02**		0.018397	0.010711	0.007008	0.003223	0.001522
**0.04**		0.036054	0.020183	0.011824	0.0059	0.002966
**0.06**		0.053316	0.029533	0.017299	0.008684	0.004425
**0.08**		0.070486	0.039309	0.021911	0.011428	0.005716
**0.1**		0.08687	0.048695	0.028106	0.014168	0.007157
**0.2**		0.170919	0.09606	0.05549	0.027996	0.014275
**0.4**		0.325024	0.186813	0.108212	0.056845	0.029569
**0.6**			0.271244	0.162753	0.086508	0.045808
**0.8**				0.212227	0.11706	0.063123
**1**				0.265064	0.147898	0.0814
k		0.808858	0.452293	0.264542	0.146865	0.080242
±		0.008135	0.003649	0.001018	0.000851	0.00104
T		343.2	337.5	331.8	326.1	320.4
1/T		0.002914	0.002963	0.003014	0.003067	0.003121
ln(k/T)		-6.05045	-6.61499	-7.13429	-7.70545	-8.29228
ln(k)		-0.21213	-0.79343	-1.32976	-1.91824	-2.52271

Figure S2: Plot of normalised intensity vs mixing time for the reaction of [Sn{O^{i}Pr}$_2$] with ^{t}BuNCO at 320.4 K, 326.1 K, 331.8 K, 337.5 K and 343.2 K respectively.

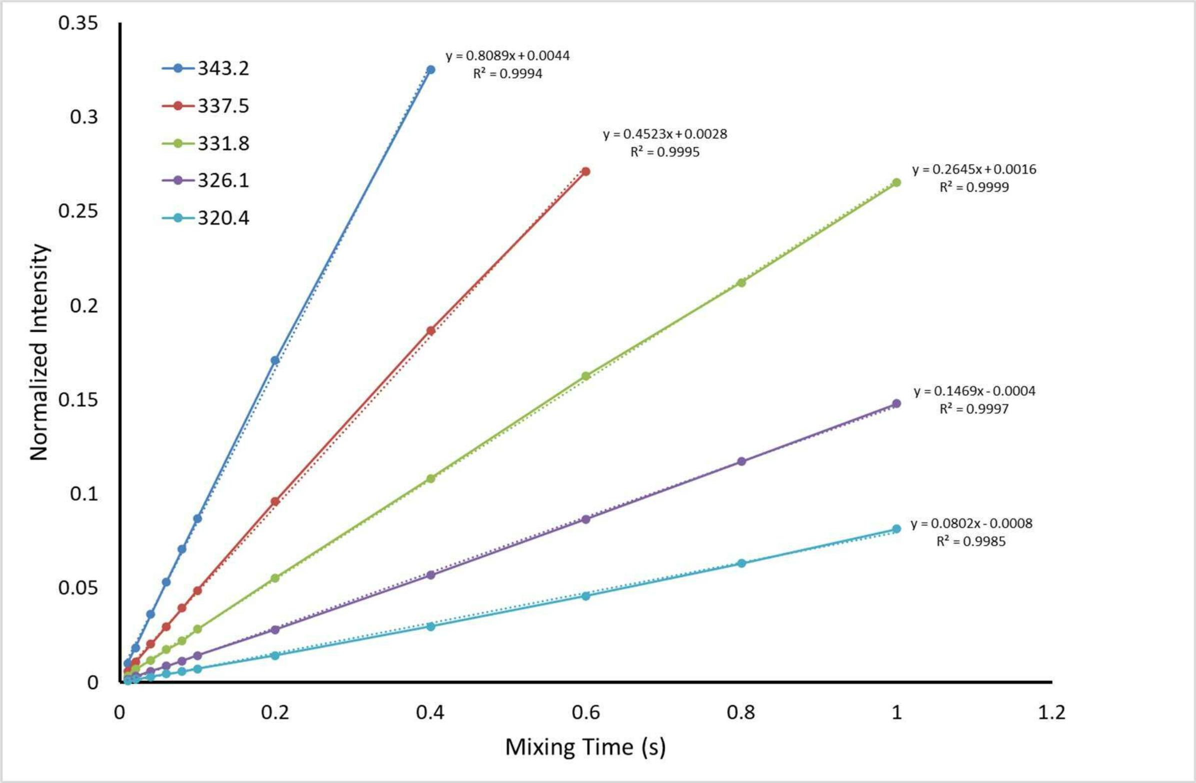

Table S4: Table of experimentally determined equilibrium rate constants at different temperatures for the reaction between [Sn{O^{i}Pr}$_2$] with tBuNCO.

Temperature (K)	*k* (s^{-1})
**320.4**	0.0802
**326.1**	0.1469
**331.8**	0.2645
**337.5**	0.4523
**343.2**	0.8089

Figure S3: An Arrhenius Plot of the experimentally determined equilibrium rate constants at different temperatures, 320.4 K, 326.1 K, 331.8 K, 337.5 K and 343.2 K respectively, for the reaction between [Sn{O^{i}Pr}$_2$] with ^{t}BuNCO.

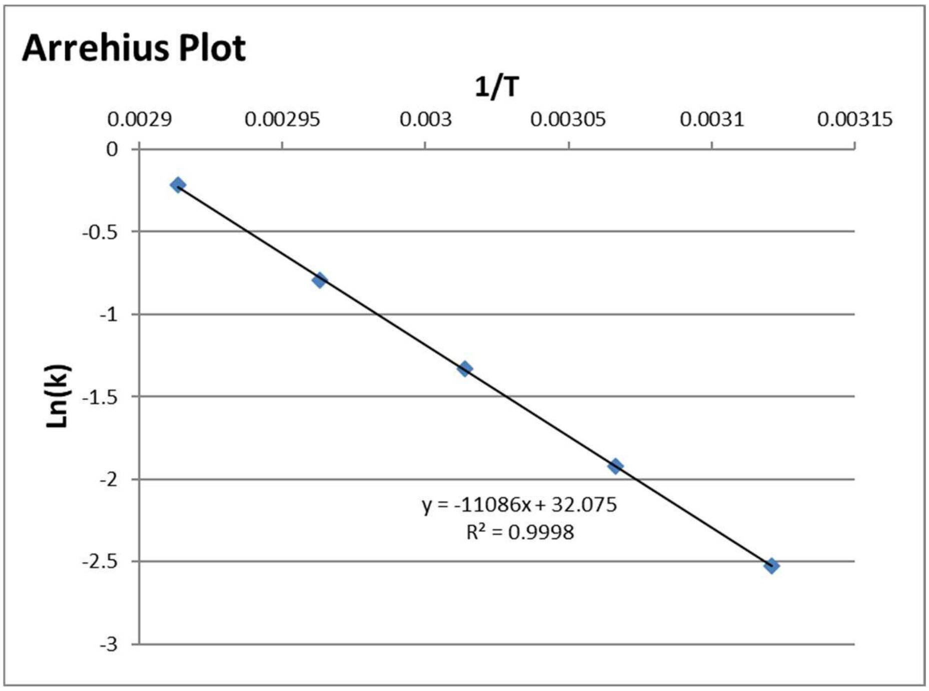

Figure S4: An Eyring Plot of the experimentally determined equilibrium rate constants at different temperatures, 320K, 326K, 332K, 338K and 343K respectively, for the reaction between [Sn{O^{i}Pr}$_2$] with ^{t}BuNCO.

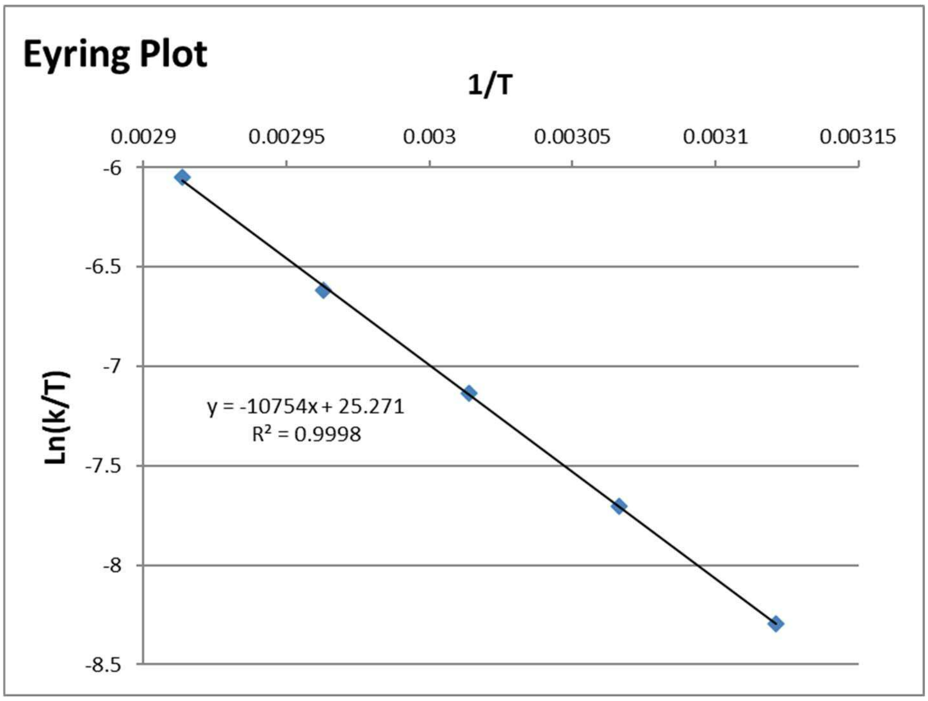

Table S5: Arrhenius and Eyring Calculation data for the reaction between $[Sn\{O^{i}Pr\}_2]$ with $^{t}BuNCO$.

Arrhenius Data:

Regression Statistics	
Multiple R	0.999891934
R Square	0.99978388
Adjusted R Square	0.999711839
Standard Error	0.015425457
Observations	5

ANOVA

	df	*SS*	*MS*	*F*	*Significance F*
Regression	1	3.302231762	3.302231762	13878.14662	1.34853E-06
Residual	3	0.000713834	0.000237945		
Total	4	3.302945596			

	Coefficients	*Standard Error*	*t Stat*	*P-value*	*Lower 95%*	*Upper 95%*	*Lower 95.0%*	*Upper 95.0%*
Intercept	32.07471643	0.283856306	112.9963146	1.52811E-06	31.17135898	32.97807388	31.17135898	32.97807388
X Variable 1	-11085.51453	94.10010755	-117.8055458	1.34853E-06	-11384.98307	-10786.04599	-11384.98307	-10786.04599

Ea	92.17009616	±	0.782391827

Eyring Data

Regression Statistics	
Multiple R	0.999886931
R Square	0.999773874
Adjusted R Square	0.999698499
Standard Error	0.015306709
Observations	5

ANOVA

	df	*SS*	*MS*	*F*	*Significance F*
Regression	1	3.107680161	3.107680161	13263.94389	1.44326E-06
Residual	3	0.000702886	0.000234295		
Total	4	3.108383047			

	Coefficients	*Standard Error*	*t Stat*	*P-value*	*Lower 95%*	*Upper 95%*	*Lower 95.0%*	*Upper 95.0%*
Intercept	25.27076474	0.281671124	89.71727165	3.05245E-06	24.37436152	26.16716797	24.37436152	26.16716797
X Variable 1	-10754.0051	93.37570635	-115.1691968	1.44326E-06	-11051.16827	-10456.84193	-11051.16827	-10456.84193

R =

8.314462618	$J \cdot K^{-1} \cdot mol^{-1}$

kb	1.38E-23			
h	6.63E-34			
23.75997748				
delta H	89.41377339	kJ mol-1	±	0.77636882
delta S	12.56138424	J mol-1 K-1	±	-2.823791198

Figure S5: ^{1}H NMR Spectrum (d^8-tol) of the 1:1 reaction of CO_2 with [Sn{O^{i}Pr}$_2$] at 298K.

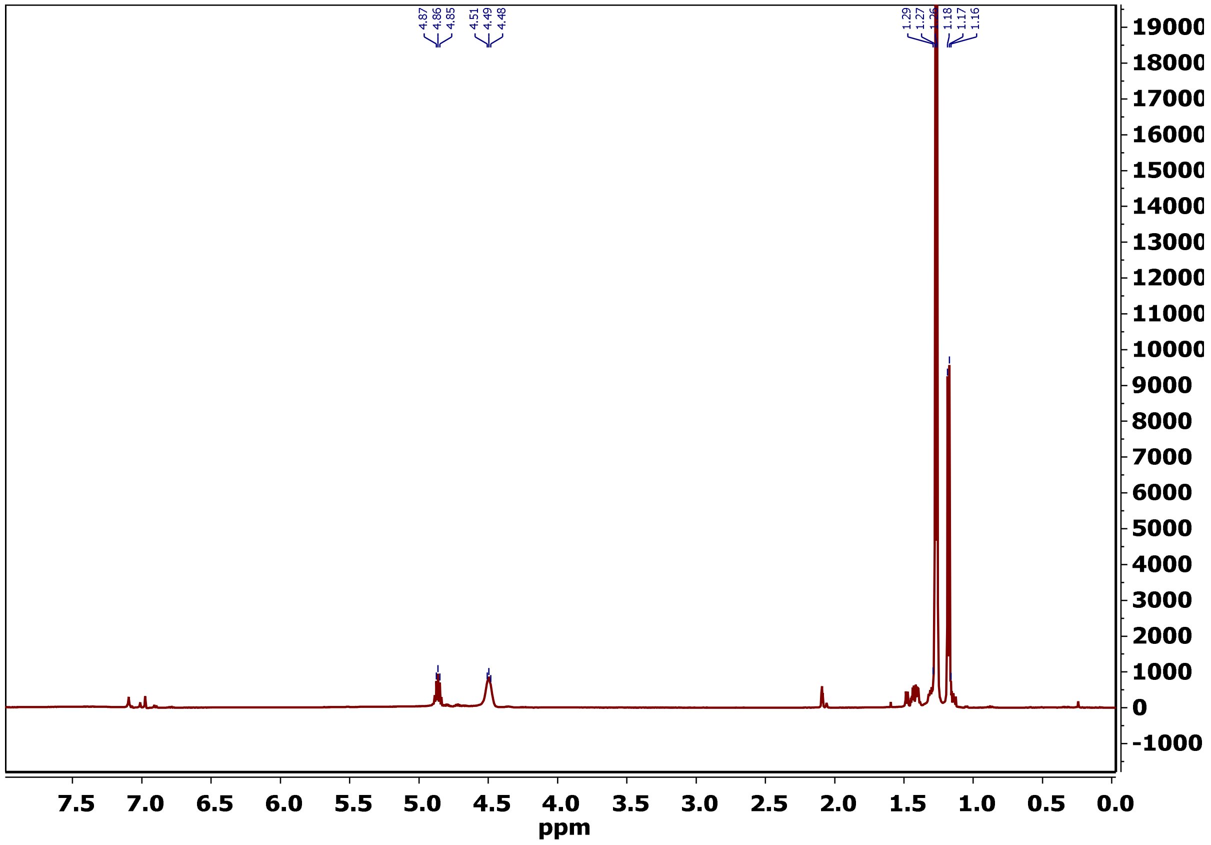

Table S6: Mixing time and normalised intensities for the reaction of $[Sn\{O^iPr\}_2]$ with CO_2 at 266.6, 271.9, 277.4, 288.0, 297.9 and 302.8 K respectively.

Temp K	**302.8**	**297.9**	**288.0**	**277.4**	**271.9**	**266.6**
Temp oC	29.65	24.75	14.85	4.25	-1.25	-6.55
	Normalized Intensity					
Mixing Time (s)						
**0.01**	0.0432	0.025	0.008395	0.003616	0.00091	0.00071
**0.02**	0.0814	0.0511	0.017123	0.005472	0.000805	0.001988
**0.04**	0.1658	0.1058	0.034869	0.012075	0.006361	0.003435
**0.06**	0.2729	0.1512	0.051781	0.017762	0.010167	0.005508
**0.08**	0.339	0.2178	0.070264	0.024713	0.013615	0.007217
**0.1**	0.4391	0.2675	0.087851	0.030914	0.017705	0.009179
**0.15**	0.6356	0.4134	0.133125	0.046321	0.027335	0.014152
**0.2**	0.8588	0.527	0.179037	0.060792	0.036673	0.018621
**0.3**					0.052422	
k	4.2867	2.6885	0.8975	0.3062	0.1829	0.0942
±	0.5115	0.041509	0.00374	0.002972	0.003906	0.000978
T	302.8	297.9	288	277.4	271.9	266.6
1/T	0.003303	0.003357	0.003472	0.003605	0.003678	0.003751
ln(k/T)	-4.25756	-4.70777	-5.7711	-6.80898	-7.30425	-7.94808
ln(k)	1.455517	0.988983	-0.10814	-1.18352	-1.69882	-2.36234

Figure S6: Plot of normalised intensity vs mixing time for the reaction of [Sn{O^{i}Pr}$_2$] with CO_2 at 266.6, 271.9, 277.4, 288, 297.9 and 302.8 K respectively.

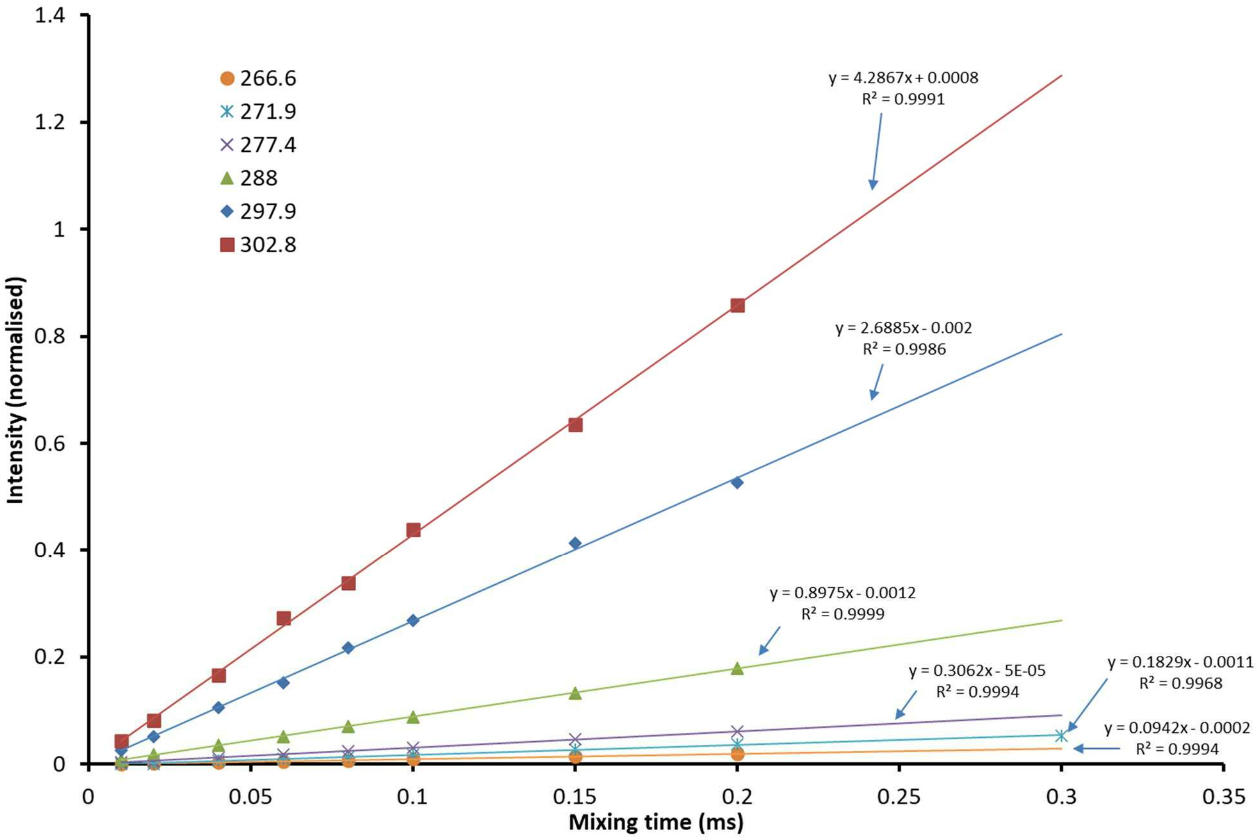

Table S7: Table of experimentally determined equilibrium rate constants at different temperatures for the reaction between [Sn{O^{i}Pr}$_2$] with CO_2.

Temperature (K)	k (s^{-1})
**266.6**	0.0942
**271.9**	0.1829
**277.4**	0.3062
**288.0**	0.8975
**297.9**	2.6885
**302.8**	4.2867

Table S8: Arrhenius and Eyring Calculation data for the reaction of [Sn{O^{i}Pr}$_2$] with CO_2.

Arrhenius Data

Regression Statistics	
Multiple R	0.99931001
R Square	0.998620495
Adjusted R Square	0.998275619
Standard Error	0.063157023
Observations	6

ANOVA

	df	*SS*	*MS*	*F*	*Significance F*
Regression	1	11.54996165	11.54996	2895.5912	7.1397E-07
Residual	4	0.015955238	0.003989		
Total	5	11.56591689			

	Coefficients	*Standard Error*	*t Stat*	*P-value*	*Lower 95%*	*Upper 95%*	*Lower 95.0%*	*Upper 95.0%*
Intercept	29.36378049	0.555293439	52.87975	7.655E-07	27.8220387	30.9055222	27.8220387	30.90552224
X Variable 1	-8461.56751	157.2469393	-53.8107	7.14E-07	-8898.155	-8024.98001	-8898.155	-8024.980012

Ea	-70.3533867	±	1.307424

Eyring Plot

Regression Statistics	
Multiple R	0.999276438
R Square	0.998553399
Adjusted R Square	0.998191748
Standard Error	0.062504787
Observations	6

ANOVA

	df	*SS*	*MS*	*F*	*Significance F*
Regression	1	10.78720677	10.78720677	2761.102	7.85124E-07
Residual	4	0.015627393	0.003906848		
Total	5	10.80283416			

	Coefficients	*Standard Error*	*t Stat*	*P-value*	*Lower 95%*	*Upper 95%*	*Lower 95.0%*	*Upper 95.0%*
Intercept	22.71311722	0.549558806	41.32973031	2.05E-06	21.18729737	24.23893708	21.18729737	24.23893708
X Variable 1	-8177.396463	155.6230167	-52.5461891	7.85E-07	-8609.475226	-7745.3177	-8609.475226	-7745.3177

R =

8.314462618	J·K^{-1}·mol^{-1}

kb	1.38E-23			
h	6.63E-34			
23.75997748				
delta H	67.99065721	kJ mol-1	±	1.293922
delta S	-8.704080486	J mol-1 K-1	±	-2.79157

1_298K_C6D6_1H

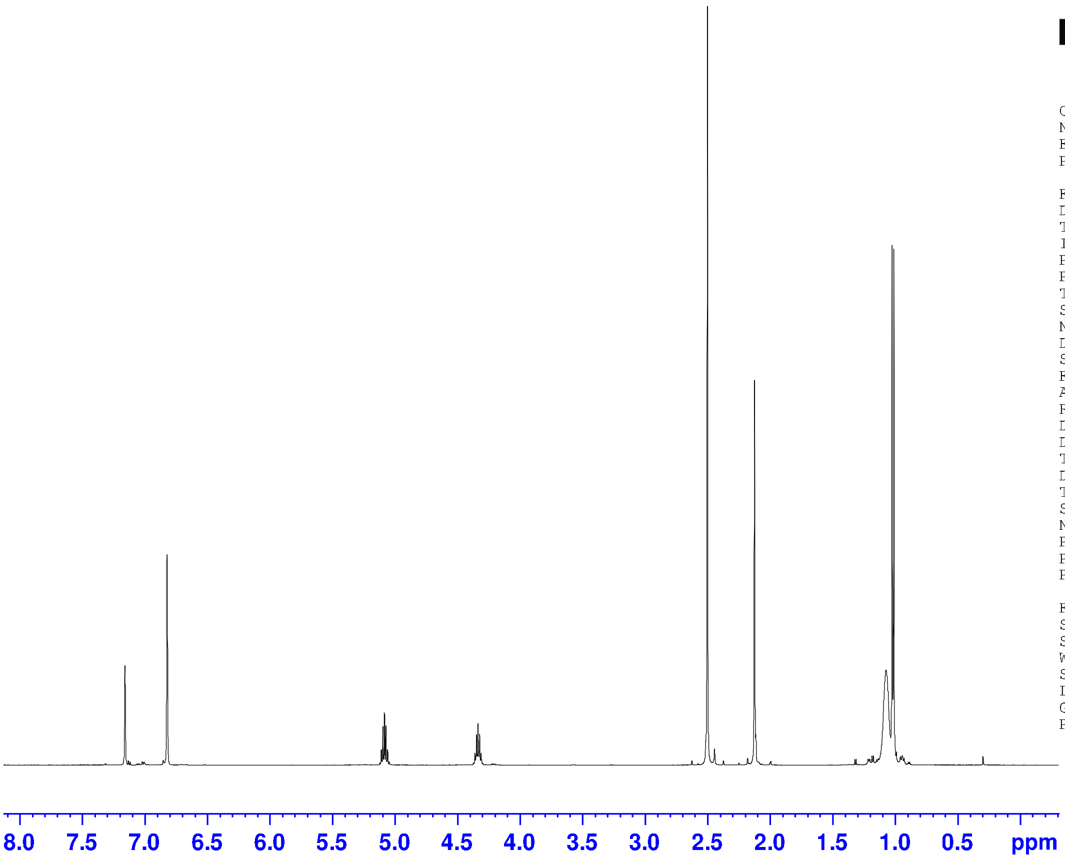


```
Current Data Parameters
NAME      24_02_15_ARYA_TW1
EXPNO                    10
PROCNO                    1

F2 - Acquisition Parameters
Date_              20240215
Time                  13.56 h
INSTRUM              Avance
PROBHD    Z186262_0001 (
PULPROG                zg30
TD                    65536
SOLVENT               CDCl3
NS                        8
DS                        0
SWH               10000.000 Hz
FIDRES             0.305176 Hz
AQ                3.2767999 sec
RG                      101
DW                   50.000 usec
DE                    11.14 usec
TE                    298.0 K
D1               1.00000000 sec
TD0                       1
SFO1            500.1330883 MHz
NUC1                     1H
P0                     2.67 usec
P1                     8.00 usec
PLW1            27.93600082 W

F2 - Processing parameters
SI                    65536
SF              500.1300358 MHz
WDW                      EM
SSB                       0
LB                     0.30 Hz
GB                        0
PC                     1.00
```

Diffusion Analysis

1_298K C6D6-1HDOSY

sample name:	1_298K_Benzene_1HDOSY
Description/Title:	
Origin:	in-house
Date of preparation:	15 Mar 2024
Lab Book Number:	000

Diffusion Analysis
C:/Bruker/TopSpin4.0.7/examdata/24_02_15_ARYA_TW1/30/pdata/1/2rr

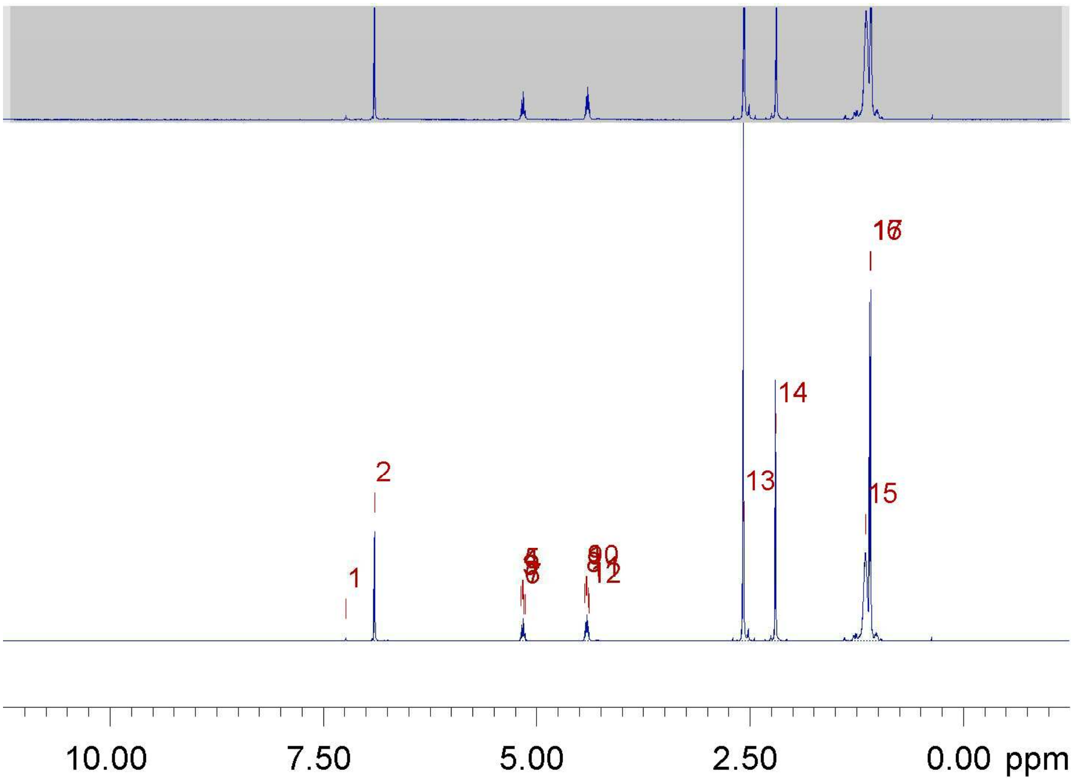

Dosy/Fit

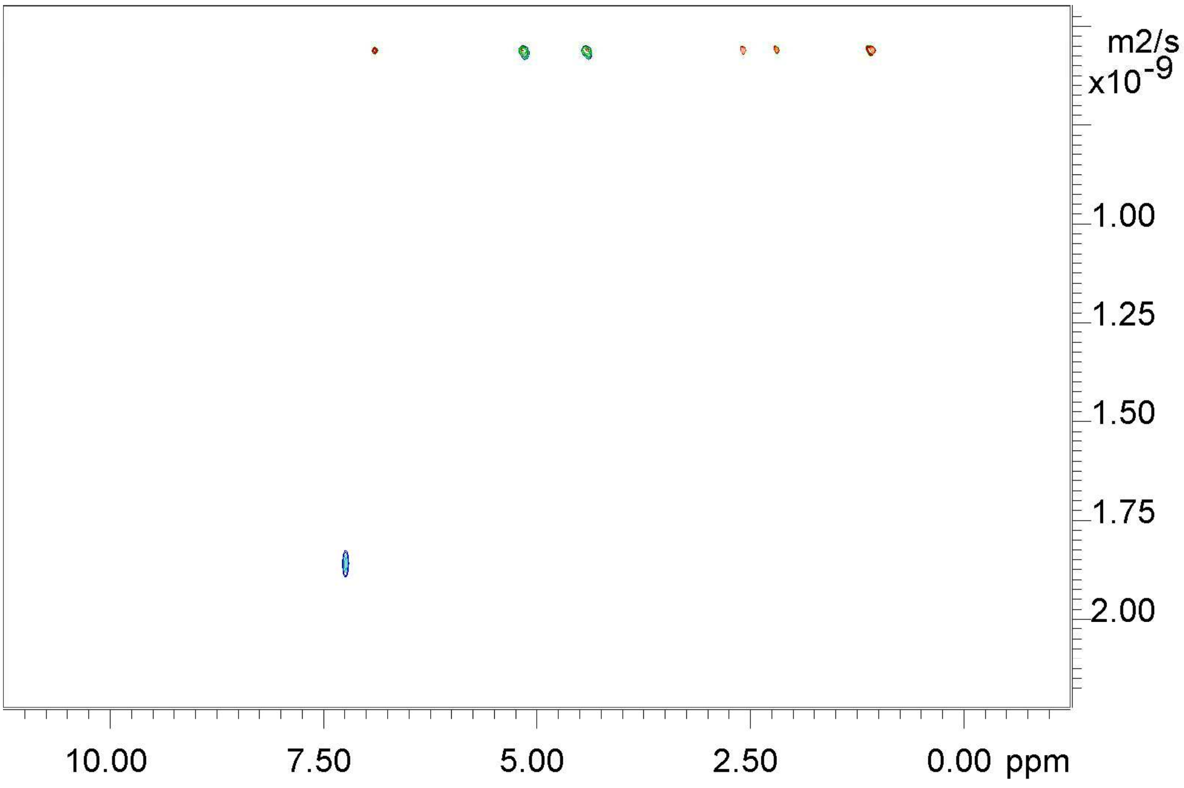

Diffusion Analysis
C:/Bruker/TopSpin4.0.7/examdata/24_02_15_ARYA_TW1/30/pdata/1/2rr

Fitted function:	f (x) = Io * exp (-D * x^2 * gamma^2 * littleDelta^2 (bigDelta-littleDelta/3)* 10^4
used gamma:	26752 rad/(s*Gauss)
used little delta:	0.0020000 s
used big delta:	0.059900 s
used gradient strength:	variable
Random error estimation of data:	RMS per spectrum (or trace/plane)
Systematic error estimation of data:	worst case per peak scenario
Fit parameter Error estimation method:	from fit using calculated y uncertainties
Confidence level:	95%
Used peaks:	peaks from C:/Bruker/TopSpin4.0.7/examdata/24_02_15_ARYA_TW1/30/pdata/1/peaklist1D.xml
Used integrals:	peak intensities
Used Gradient strength:	all values (including replicates) used

Peak name	F2 [ppm]	D [m2/s]	error	fitInfo
1	7.231	1.86e-09	2.033e-11	Done
2	6.894	5.62e-10	1.613e-13	Done
3	5.181	5.56e-10	2.804e-12	Done
4	5.167	5.57e-10	1.100e-12	Done
5	5.155	5.60e-10	7.892e-13	Done
6	5.143	5.65e-10	1.014e-12	Done
7	5.130	5.70e-10	2.399e-12	Done
8	4.430	5.55e-10	2.323e-12	Done
9	4.418	5.59e-10	9.222e-13	Done
10	4.406	5.62e-10	6.757e-13	Done
11	4.394	5.65e-10	8.871e-13	Done
12	4.381	5.69e-10	2.158e-12	Done
13	2.571	5.62e-10	3.407e-14	Done
14	2.194	5.65e-10	6.796e-14	Done
15	1.143	5.57e-10	1.996e-13	Done
16	1.092	5.64e-10	5.227e-14	Done
17	1.080	5.66e-10	5.056e-14	Done

1_298K_C6D6_119Sn

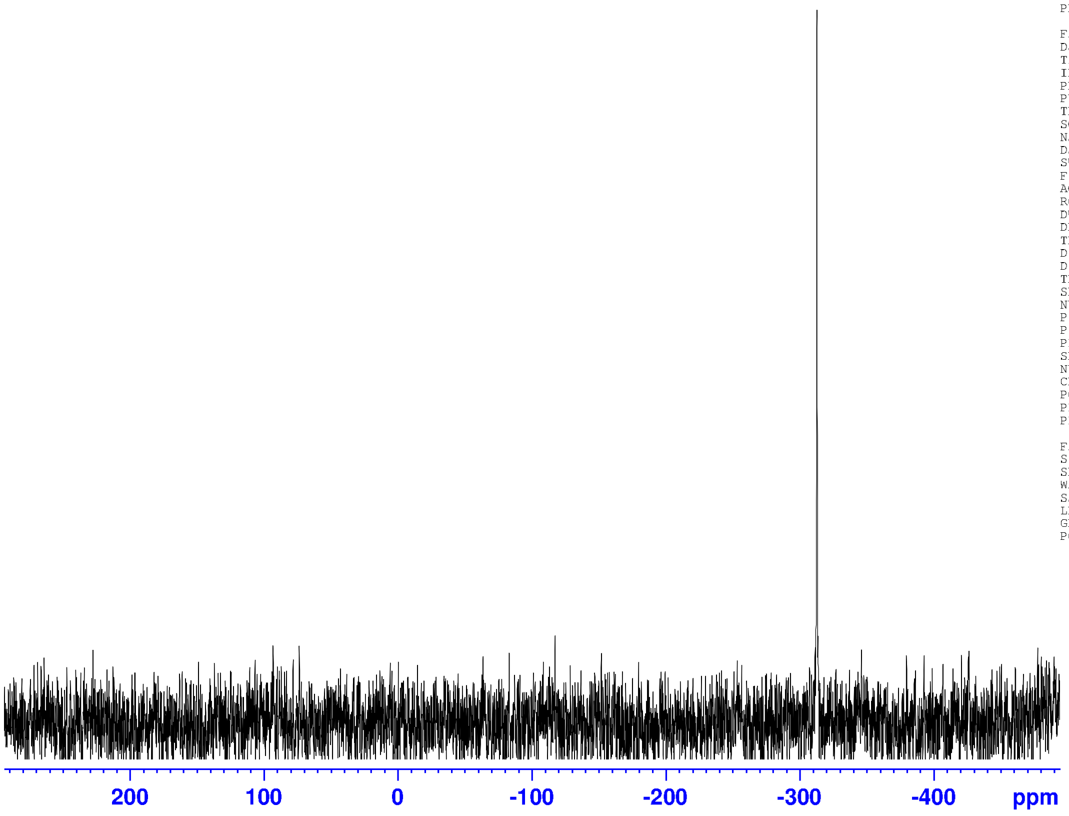


```
Current Data Parameters
NAME      24_02_27_ARYA_TW1_RTand350K
EXPNO                     12
PROCNO                     1

F2 - Acquisition Parameters
Date_            20240227
Time                10.18 h
INSTRUM            Avance
PROBHD    Z186262_0001 (
PULPROG           zgig30
TD                 65536
SOLVENT             C6D6
NS                   258
DS                     0
SWH           147058.824 Hz
FIDRES          4.487879 Hz
AQ             0.2228224 sec
RG                   101
DW                 3.400 usec
DE                  6.50 usec
TE                 298.0 K
D1            1.00000000 sec
D11           0.03000000 sec
TD0                    1
SFO1         186.4829877 MHz
NUC1               119Sn
P0                  4.00 usec
P1                 12.00 usec
PLW1         80.00000000 W
SFO2         500.1320005 MHz
NUC2                  1H
CPDPRG[2         waltz16
PCPD2              80.00 usec
PLW2         27.93600082 W
PLW12         0.27936000 W

F2 - Processing parameters
SI                 32768
SF           186.5016378 MHz
WDW                   EM
SSB                    0
LB                 20.00 Hz
GB                     0
PC                  1.40
```

1_350K_C6D6_1H

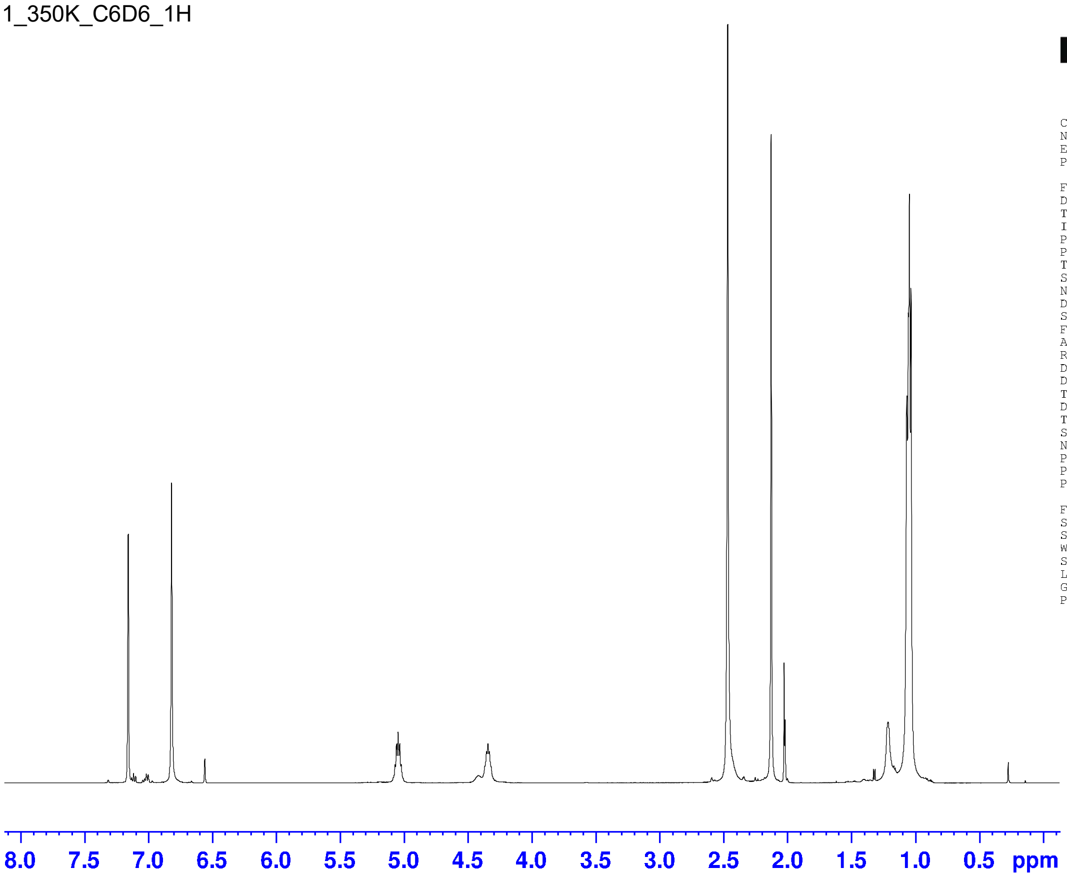


```
Current Data Parameters
NAME     24_02_27_ARYA_TW1_RTand350K
EXPNO                     40
PROCNO                     1

F2 - Acquisition Parameters
Date_             20240227
Time                 11.27 h
INSTRUM             Avance
PROBHD   Z186262_0001 (
PULPROG               zg30
TD                   65536
SOLVENT               C6D6
NS                       8
DS                       0
SWH              10000.000 Hz
FIDRES            0.305176 Hz
AQ               3.2767999 sec
RG                     101
DW                  50.000 usec
DE                   11.14 usec
TE                   349.2 K
D1              1.00000000 sec
TD0                      1
SFO1           500.1330883 MHz
NUC1                    1H
P0                    2.67 usec
P1                    8.00 usec
PLW1           27.93600082 W

F2 - Processing parameters
SI                   65536
SF             500.1299961 MHz
WDW                     EM
SSB                      0
LB                    0.30 Hz
GB                       0
PC                    1.00
```

Diffusion Analysis

1_350K_C6D6_1HDOSY

sample name:	1_350K_Benzene_1HDOSY
Description/Title:	
Origin:	in-house
Date of preparation:	15 Mar 2024
Lab Book Number:	000

Diffusion Analysis
C:/Bruker/TopSpin4.0.7/examdata/24_02_27_ARYA_TW1/41/pdata/1/2rr

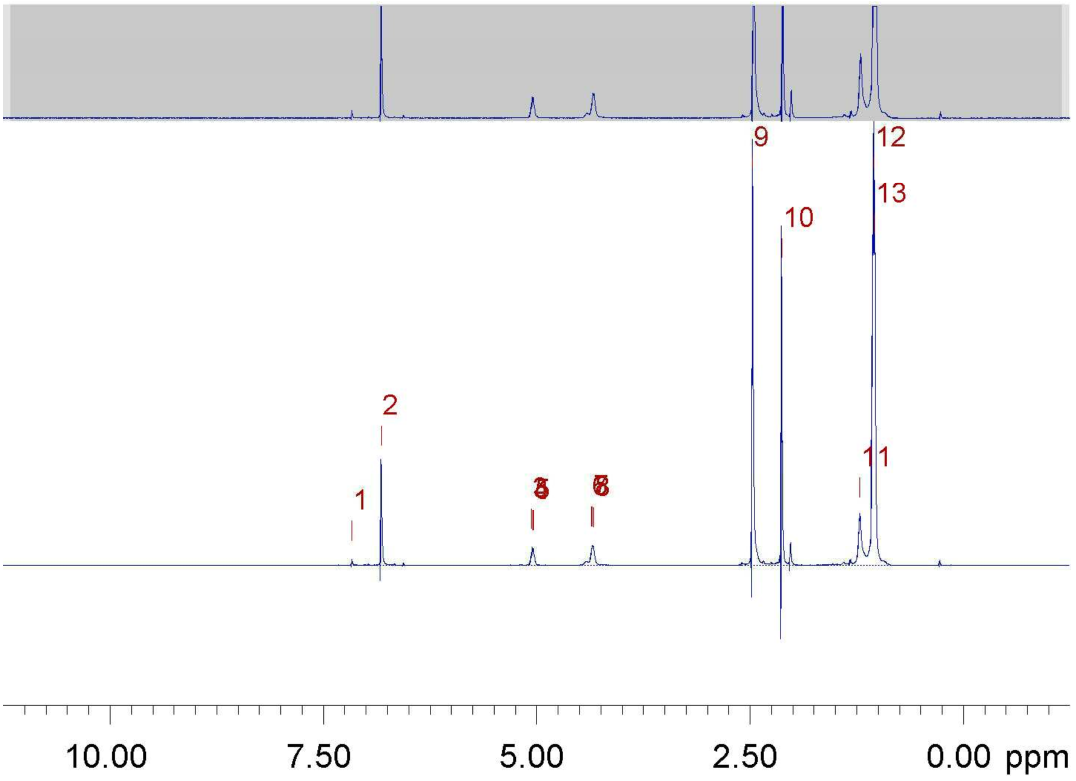

Dosy/Fit

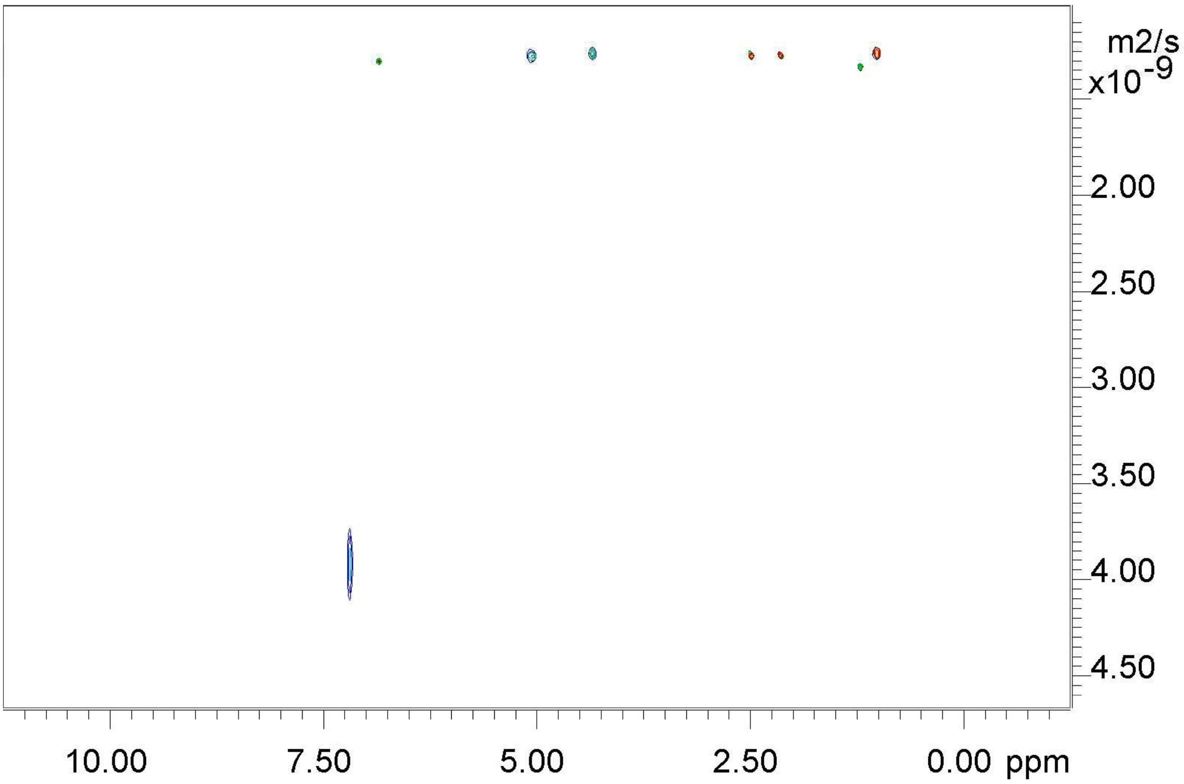

Diffusion Analysis
C:/Bruker/TopSpin4.0.7/examdata/24_02_27_ARYA_TW1/41/pdata/1/2rr

BRUKER

Fitted function:	f (x) = Io * exp (-D * x^2 * gamma^2 * littleDelta^2 (bigDelta-littleDelta/3)* 10^4
used gamma:	26752 rad/(s*Gauss)
used little delta:	0.0020000 s
used big delta:	0.056730 s
used gradient strength:	variable
Random error estimation of data:	RMS per spectrum (or trace/plane)
Systematic error estimation of data:	worst case per peak scenario
Fit parameter Error estimation method:	from fit using calculated y uncertainties
Confidence level:	95%
Used peaks:	peaks from C:/Bruker/TopSpin4.0.7/examdata/24_02_27_ARYA_TW1/41/pdata/1/peaklist1D.xml
Used integrals:	peak intensities
Used Gradient strength:	all values (including replicates) used

Peak name	F2 [ppm]	D [m2/s]	error	fitInfo
1	7.161	3.91e-09	1.871e-10	Done
2	6.818	1.30e-09	2.872e-12	Done
3	5.059	1.28e-09	2.607e-11	Done
4	5.047	1.28e-09	1.703e-11	Done
5	5.034	1.29e-09	1.800e-11	Done
6	4.355	1.25e-09	2.149e-11	Done
7	4.343	1.27e-09	1.453e-11	Done
8	4.333	1.28e-09	1.505e-11	Done
9	2.467	1.27e-09	6.908e-13	Done
10	2.127	1.28e-09	8.704e-13	Done
11	1.207	1.34e-09	6.073e-12	Done
12	1.045	1.27e-09	6.597e-13	Done
13	1.033	1.26e-09	7.528e-13	Done

References

1. O. G. Shirobokov, S. I. Gorelsky, R. Simionescu, L. G. Kuzmina, and G. I. Nikonov, Chem. Commun., 2010, 46, 7831–7833
